# Fostering inclusive science media: Insights from examining the relationship between women’s identities and their anticipated engagement with *Deep Look* YouTube science videos

**DOI:** 10.1371/journal.pone.0308558

**Published:** 2024-08-09

**Authors:** Jocelyn Steinke, Christine Gilbert, Kelsi Opat, Asheley R. Landrum

**Affiliations:** 1 Department of Communication, University of Connecticut, Storrs, Connecticut, United States of America; 2 School of Communication & Journalism, Stony Brook University, Stony Brook, New York, United States of America; 3 College of Media and Communication, Texas Tech University, Lubbock, Texas, United States of America; 4 Department of Agricultural Leadership, Education, and Communications, Texas A & M University, College Station, Texas, United States of America; 5 Walter Cronkite School of Journalism and Mass Communication, Arizona State University, Phoenix, Arizona, United States of America; SEGi University Kota Damansara, MALAYSIA

## Abstract

As science media content creators strive for inclusivity in communication design and promotion, they must consider the influence of audiences’ identities on their engagement with science media. A gender gap in viewership or "missing audience" has been identified for women viewers for educational science content on digital media; one such example of this is *Deep Look*, a science video series from KQED public media and PBS Digital Studios distributed on YouTube. This study used a mixed method design (1) to examine women’s preferences for *Deep Look* YouTube video promotions (i.e., episode titles and thumbnail images—the images that act as a preview for the video) to best inform future design of promotional content for these videos to attract more women viewers, and (2) to explore how women’s preferences for science content are linked to their social identities, science identity, and science curiosity. Findings indicated that women’s preferences for promotions for *Deep Look* YouTube science and nature videos followed expected trends with most women preferring images perceived as visually attractive and colorful more than images perceived as disgusting or gross. However, these preferences were conditional on science curiosity and science identity. Findings indicated that to boost women’s engagement with YouTube science and nature videos, content creators may find it useful to consider how science curious various women audiences are, how strongly women viewers identify with being a science person, and how their most salient social identities motivate engagement.

## Introduction

Online media have become primary sources of information about science, technology, and the environment [1, p. 31]. Yet, science communication audiences vary by gender with the most engaged audiences, *e*.*g*., “Sciencephiles” and “Critically Interested,” being comprised primarily of men [2], and the least engaged audiences, *e*.*g*., “Passive Supporters” and “Disengaged,” being comprised mostly of women [2]. In describing demographic characteristics of the segmented audiences of science communication (i.e., “Sciencephiles,” “Critically Interested,” “Passive Supporters,” and “Disengaged”), Schäfer et al. [2] operationalize gender using a binary categorization (male and female). Knowledge of factors that best promote women’s engagement with online science media is needed to identify best practices for *inclusive* science communication. First described as “socially inclusive science communication” [3], inclusive science communication has been defined as “how social scientific and rhetorical approaches can be used to increase inclusivity in public engagement practice” [4]. While women and girls have been the most studied audience for inclusive science communication research in the past 40 years [5], little research has focused on identifying how specific media content features and women viewers’ characteristics influence their science engagement for online science media.

Prior research has identified a gender gap or a “missing audience” of women viewers for YouTube science and nature videos [6, 7]. And while YouTube is an enormously popular platform with an estimated 2.5 billion global unique users a month [8] and continues to dominate the online landscape with 81% of U.S. adults reported in 2021 [9] and approximately 90% of U.S. teens in 2023 [10], research has suggested that understanding YouTube as a science communication tool is still in its infancy [11, 12].

*Deep Look*, a YouTube series produced by the San Francisco-based, public media outlet KQED, seeks to promote public interest and knowledge about nature and science through videos focusing on bugs and insects, small animals, sea life, and plants, and it releases episodes twice each month on https://www.youtube.com/@KQEDDeepLook. The series has a female narrator and uses macro photography and microscopy in 4K resolution to see science “up close”. The current project focused on science engagement for women because YouTube analytics available on (binary) gender indicate that women are not as engaged with Deep Look YouTube videos as men. Although the Deep Look channel has over 2.2 million subscribers, its viewership is disproportionately male: YouTube metrics for the series, available to the producers, have indicated a total viewership comprised of approximately 70% men and 30% women, with over 90% male viewers for some individual episodes. This gender disparity mirrors the reported disparities of similar digital science shows [7] and was replicated in an online experiment that aimed to circumvent any potential effects of the YouTube algorithm [6]. Thus, it underscores a need for closer investigation of gender differences in science engagement.

While conceptualizations of science engagement have varied widely [13], a recent conceptualization described science engagement for YouTube science content as emotional, cognitive, and behavioral manifestations of connection and involvement with YouTube content [14]. In this work, science engagement was operationalized as behavioral engagement online (e.g., liking, disliking, commenting), which was connected to expressions of emotional engagement (e.g., anger, anticipation, disgust, fear, joy, sadness, surprise, and trust) in comments posted online, which in turn, prompted cognitive engagement (argumentative deliberation) in comments posted online [14]. However, this conceptualization described science engagement for those already actively engaging with online science media content. It ignores the “missing audiences”—those who might be interested in engaging with science media, but for some yet undiscovered reason are not [15]—as well as the less interested and even disinterested science media audiences [2, 16]. More research is needed to identify best practices for designing online science media for these audiences. To accomplish this, research needs to examine engagement during the initial and early stages of exposure to predict whether deeper engagement can even be achieved for more *tentative* science media audiences. In addition, design features of science media content need to be carefully considered to determine which features best promote science engagement for all audiences.

The present research examined women’s science engagement during their initial exposure to science content featured in promotions for *Deep Look* YouTube science and nature videos. This research used a mixed methods two-part study to 1) assess ***what*** promotions (episode titles and thumbnail images—the images that act as a preview for the video) women preferred and 2) explore the reasons ***why*** women reported engagement or lack of engagement when viewing promotions for *Deep Look* videos. For the first study, a survey and natural language processing (NLP) analysis of open-ended survey responses examined ***what*** content prompted women’s engagement (preferences, reasons for preferences, intent to view) and examined women’s reported preferences as related to their science curiosity [17], science identity or seeing themselves as a “science person” [18, p. 3], and other social identities. For the second study, semi-structured interviews investigated ***why*** women’s preferences were influenced by their science identity and other social identities. The additional use of this qualitative approach helped provide insights to better understand ***how*** women’s specific social identities might have influenced their science media engagement and also amplified the voices describing specific needs of this underserved science communication audience. This research used social identity theory as a framework to consider how women’s most central identities influenced engagement during their exposure to digital science video promotions and to examine how social identities influenced women’s engagement with online science media.

### Social identity theory

Identity has been defined as one’s “core sense of self”[19, p. 405] and as one’s “internal self-constructed, dynamic organization of drives, abilities, beliefs, and individual history” [20, p. 159]. According to Stets and Burke [21], self-identity or person identity can be defined as “the categorization of the self as a unique entity, distinct from other individuals” (p. 228). Although self-identity often is referred to as a single entity, according to Korte [22], self-identity includes both core concepts of self, which are more enduring, as well as peripheral concepts of self, which are more fluid and dynamic. Patterson and colleagues [23] describe the ongoing nature of self-identity development, which changes in relation to group identities or social identities. Thus, self-identity is dynamic and reflexive as individuals take an ongoing active role in identity formation as they strive to associate with groups they perceive as most desirable [24]. Further, individuals may engage in identity switching, drawing on a particular group identity that may be activated at different times and in different contexts, and thus, made more salient [25].

Social identity theory [24] provides a strong theoretical framework for this research because it acknowledges how individuals’ identification and association with social groups [24, 26] drive and motivate behavior [27]. Social identity theory recognizes that individuals simultaneously identify with multiple social groups, which are selected based on which identities are perceived to be most desirable and most important for maintaining a positive sense of self [24, 28]. Social identity theory explains that “individuals tend to favour those whom they see as members of their own group (‘in-group’) over those they see as belonging to another group (‘out-group’)” [29, p. 318]. Identity is a complex construct because identities are multi-dimensional; individuals simultaneously associate with multiple different social groups [18, 24]. Social identities may include gender, race, ethnicity, sexuality, ability, family roles, political affiliation, occupation, religion, as well as other social group associations. Overall, social identity theory is helpful in understanding how social identities influence people’s motivations for attending to information, including media content, best aligned with their desired social identities.

Individuals actively enact identities that are most salient for a particular context or situation [29, 30]. Individuals are not bound to one group identity, and they may move from one to another more salient or desirable group identity depending on context [30]. For example, if a woman identifies as a ‘mother,’ ‘cyclist,’ and ‘scientist,’ that woman may predominately identify as a ‘scientist’ in the lab among colleagues, a ‘mother’ when surrounded by family and children, and a ‘cyclist’ when performing in a race. Previous studies have considered how underserved science communication audiences’ attitudes toward science communication and emotions correspond to feelings of inclusion in science communication [31], but little research has examined how underserved audiences’ social identities might influence inclusion in science communication. Women’s inclusion in science communication–or whether or not women perceive science communication and science media as something relevant for themselves [31, 32]–may be tied to their social identities. Thus, it is important to consider whether the framing and design used in science communication offers points of access aligned with women’s most central social identities. Subsequently, this study used social identity theory as a framework to better understand *how* women’s most central social identities and STEM (science, technology, engineering, and mathematics) identity affect their initial interest and science engagement with online science media.

### Conceptualizing science engagement

Science engagement has significance in multiple disciplines; thus, the extant literature has been vast and conceptualizations often disparate [13]. In the educational sciences, science engagement has typically been used to predict student interest and achievement in science classes [33–35]. Scholars in this area have defined science engagement as consisting of behavioral, emotional, and cognitive factors that may include or be synonymous with interest, enjoyment, motivation, and achievement in science activities in academic contexts [33, 34, 36, 37]. In the communication discipline, science engagement has been defined as communication about scientific or technical subjects that involves scientists, stakeholders, and citizens as well as science outreach focused on promoting, among other attitudes, the public understanding of science by various communicators (e.g., scientists, museum professionals, media professionals, etc., [38]). Some communication scholars have extended extant conceptualizations, suggesting that science engagement should extend to involvement in science policy issues [39] and decision making [40]. For example, research on public engagement with science communication in Japan focused on engagement as related to science policy [41]. Similarly, research in Europe segmented audiences into clusters that varied by level of participation in science: disengaged, aware, invested, and proactive [42]. Conceptualizations of public science engagement in the U.S. originated from conceptualizations of scientific literacy [43], which also considered public involvement in science policy, when identifying these audiences or publics: nonattentive, attentive, policy leaders, decision makers. The focus on advancing scientific literacy and public understanding of science often is reflected in current definitions of public engagement, with a shift from a “deficit model” to a “dialogue model” of communication between science experts and public nonexperts [38]. In the U.S., science engagement has been defined more recently as “intentional, meaningful interactions that provide opportunities for mutual learning between scientists and members of the public” [44]. More broadly, science engagement has been conceptualized to include public “attitudes toward science or desire to participate in future science activities such as attending a science fair or museum” [5, p. 12]. In prior work conducted by researchers in collaboration with KQED science communicators, science engagement was determined based on assessments of participants’ interest, surprise, attention, and how informative they found a particular YouTube video [6]. For the present study, we conceptualized science engagement as participants’ initial emotional responses to as well as their expression of behavioral intention to take future action (i.e., watch a video) when viewing promotional information (thumbnails and episode titles) for online science and nature videos.

### Gender and science identity

Prior research has described science engagement as highly gendered and linked to traditional, cultural views that historically have associated science as masculine, thus creating “a tension between science and femininity” [45 p. 147] and further supporting masculine defaults, a form of cultural bias that associates specific characteristics or behaviors with the male gender role [46]. Gender disparities in science engagement may subconsciously be connected with long-held, gendered perceptions of science and STEM subjects (science, technology, engineering, mathematics) often developed during childhood from gendered stereotypes prevalent in, and conveyed widely through, popular culture and media [47]. Prior research found that when traditional gendered stereotypes of science endorse views of science as more appropriate topics and careers for men [48], women who associate science with masculinity and identify as feminine, may experience incompatibility with their identity as a woman and identity as a scientist [49]. Indeed, consideration of identity has illuminated how cultural production and reproduction of gender and cultural representations of gender and science affect interest and persistence in science [50].

Conceptualizations of a science or STEM identity have evolved over time, emerging from an early conceptualization of identity as being recognized “as a certain ‘kind of person’” [51, p. 99]. Early conceptual frameworks described science or STEM identity as one’s representation of one’s self as a scientist or STEM professional [52; 53]. Science identity has also been described as viewing one’s self, more generally, as “a science person” [18]. Recent conceptualizations of science identity focus on identity “as a process of becoming” [50, p. 11], thus rejecting static notions of science identity and acknowledging the potential for promoting and advancing identity work [50]. These conceptualizations, then, highlight the importance of considering identity as essential for inclusive science communication. However, studies focused on gender and science identity have mostly centered on science students [18, 54, 55] or professional women scientists [49, 52, 56–58] with fewer studies focused on women who are nonexperts in science [59]. There is a significant gap in research on how (nonexpert) women’s identities, including their science identity, influence their engagement with science media content. Understanding the influence of women’s identities on intention to engage with science media content is important for determining which features of science media content are most likely to promote interest for women viewers. Prior research has noted that science communication design should recognize that target audiences differ, and, subsequently, science communicators should use different aims, messages, and channels [2].

### Current study

This study extends prior research by focusing on science engagement as related to interest in science communication [60], focusing on characteristics of promotions for online science videos intended to spark interest and drive audiences to that content. Specifically, we operationalized *anticipated* science engagement by asking participants whether they would select to watch any of several listed episodes based on the promotional information (i.e., the thumbnail and episode title). We had two research questions. First, to examine women’s preferences for *Deep Look* YouTube video promotions (thumbnails and titles) and obtain baseline data about their preferences, we asked:

RQ1: What are women’s preferences for *Deep Look* video thumbnails and titles? That is, (a) which *Deep Look* video thumbnails and titles are most and least preferred by women; (b) what are the reasons for these preferences; and (c) how might these preferences vary among women based on their science curiosity and science identity?

Second, to determine how women’s most central social identities influence their science engagement intentions and to determine which identities they cited as related to their preferences for *Deep Look* YouTube video promotions, we asked:

RQ2: How are women’s preferences for *Deep Look* YouTube video thumbnails and titles associated with their central social identities? That is, (a) which social identities do women report as their most central identities; (b) which *Deep Look* video titles and thumbnails are most and least compatible with their central social identities; and (c) what reasons did women provide to describe why their *Deep Look* video thumbnails and titles preferences were either compatible or incompatible with their most central identities?

To investigate these questions, we used an explanatory sequential (quantitative to qualitative) mixed-methods approach that included two studies: a national survey and semi-structured interviews. Explanatory sequential design can be used to “explain initial quantitative results” or to “use quantitative results about participant characteristics to guide purposeful sampling for a qualitative phase” [61 p. 77].

## Study 1: National survey

### Method

#### Participants

Survey participants (N = 1,940) were recruited by Qualtrics Research Services using quota sampling to approximate U.S. national representativeness between May 20, 2021, and June 14, 2021. See S1 Appendix. To participate in the study, participants had to be at least 18 years of age, live in the United States, and identify as female. We intentionally only recruited participants who self-identified as women because binary gender data available to the show’s producers from YouTube indicated that a much lower proportion of women than men watch KQED’s science and nature videos. At the time of data collection, YouTube did not provide information (or no information was available) about more diverse gender categories. Furthermore, our goal was to better understand *women’s* (lack of) engagement, specifically, in order to identify ways of providing greater access to science media content for this particular audience, and not to compare men’s versus women’s engagement. Participants completed the survey online and received compensation specified by survey panel agreements.

The study was approved by the Institutional Review Board at the University of Connecticut (X21-0062) and the full grant-funded project was approved by the Institutional Review Board at Texas Tech University (IRB2018-531). Written consent was obtained for the online survey, and both written and oral consent were obtained for the interviews. Survey data was de-identified and analyzed anonymously.

#### Procedure

**Fig 1:**
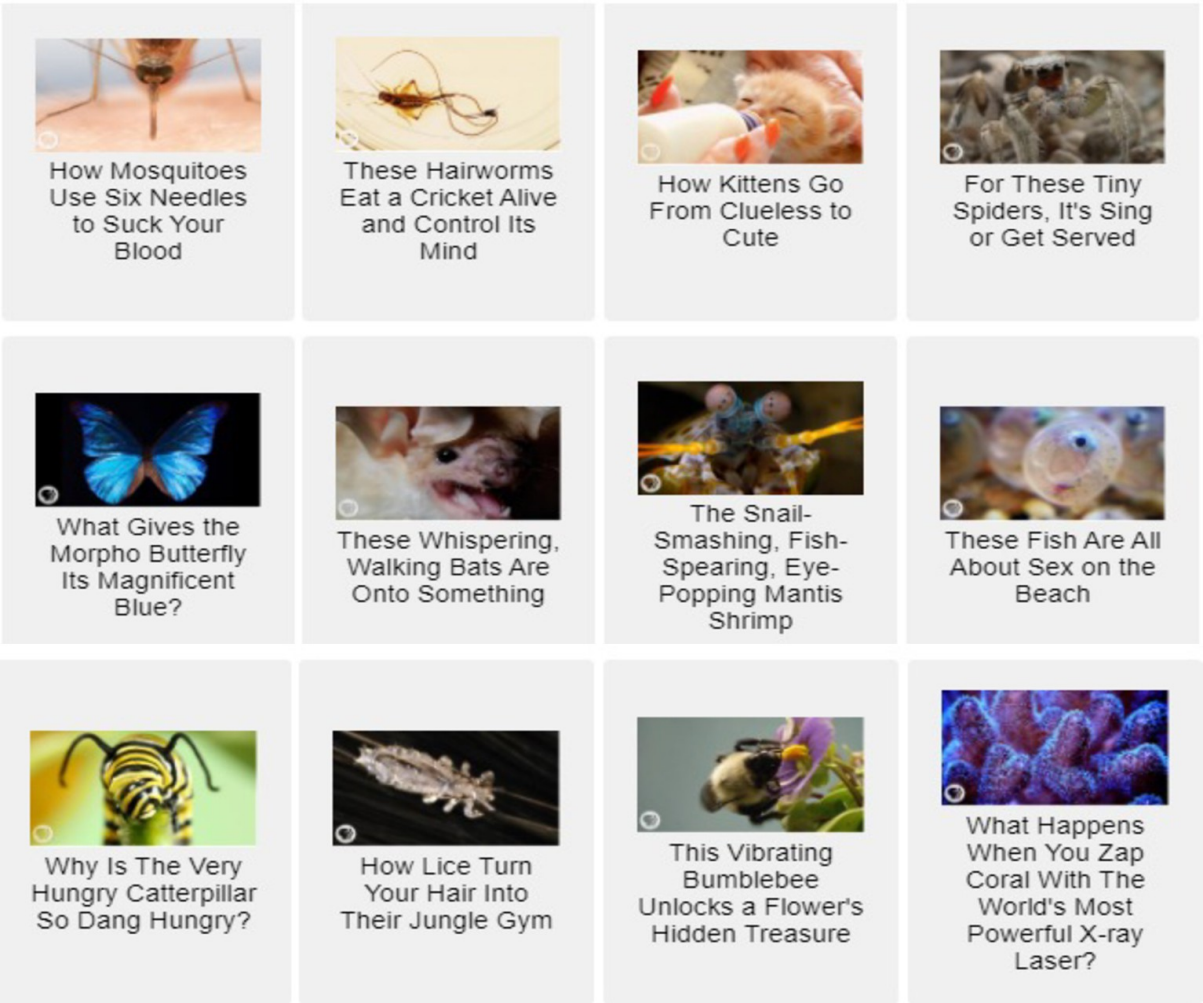
*Deep Look* YouTube video thumbnails and titles selected for the survey. Participants read the following instructions, “Below are images and titles from YouTube videos, please click on the image(s) of videos you would watch. You may select as many as you would like”.

The online survey included open- and close-ended questions related to featured thumbnails and titles for 12 *Deep Look* YouTube videos (see Fig 1). Video thumbnails and titles were selected in consultation with *Deep Look* producers based on audience preference metrics and variations in design and content related to (1) color(s) of the image in the thumbnail; (2) habitat of the animal/insect (land, water, or air); and (3) YouTube data reporting the number of female viewers for each *Deep Look* episode (e.g., the episodes watched most and least by Deep Look’s existing female audiences). The researchers determined final selection of videos.

Note that although we chose the videos based, in part, on the viewership statistics of Deep Look’s existing female audience, we did not expect these preferences to mirror the preferences of a national sample of women. This is because we do not believe that *Deep Look’s* current female audience is representative of all women or of all potential female viewers. The ultimate goal of research aiming to broaden engagement with this and similar science media (among women) is to have an audience that is representative of all women.

Although the project team, which included *Deep Look* producers and KQED Science executives, proposed the focus on the role of women’s identities in engagement, the KQED team members (including the *Deep Look* producers) were not involved in data collection, analysis, or interpretation of research findings. The median survey completion time was 19 minutes.

*Measures*. For preliminary analyses and statistics related to scale construction and evaluation (for the following measures), please see the S2 Appendix.

#### Social identity

To prime participants’ most central identities, they were asked first to list four words that describe what they consider to be their most important identities. We were interested in assessing participants’ identity using a prior conceptualization of identity that described identity as “one’s core sense of self” [19 p. 405]. Specifically, we were interested in how participants self-identified when answering the question “Who am I?” Thus, we instructed participants to use nouns that define who they are as opposed to adjectives that describe personality traits or characteristics. Participants were given an example that used Snoopy, the *Peanuts* comic character, to describe the types of words that would or would not describe Snoopy’s identities. For instance, correct examples (nouns) would be: dog, beagle, friend, companion, pilot. Incorrect examples (adjectives) would be: nice, friendly, mischievous, funny, creative. Most participants provided nouns (e.g., mom, teacher, friend, gardener, leader), although some did use adjectives (e.g., dependable, bold, caring), and others used non-identity related nouns (e.g., faith, heart). Only participant responses that used nouns to describe identity were analyzed. This approach provided a snapshot or measure of identity at a particular moment of time [50]. Then, participants were asked which two of the identities they had listed were most central to who they are or best represent their identity.

#### Preferences for video thumbnails and titles

After answering the identity questions, participants were asked to indicate their preferences for the 12 featured thumbnails and titles featured in promotions for *Deep Look*’s YouTube videos (see Fig 1). Preferences were assessed in three ways. First, participants were asked to **select** which videos they would watch (if any) for the thumbnails and titles presented. They could select as many as they would like. Second, participants were asked to **rank** the thumbnails and titles for *Deep Look* videos from one (most likely to watch) to 12 (least likely to watch). For this question, the starting order in which the thumbnails were displayed was randomized between participants. Third, participants were asked to **rate** their most-liked thumbnail and title and their least-liked thumbnail and title (based on their responses to the ranking question), using 10 semantic differential scales (Uninteresting/Interesting, Simple/Complex, Irrelevant/Relevant, Ugly/Beautiful, Disgusting/Charming, Not colorful/Colorful, Dull/Exciting, Unfamiliar/Familiar, Unlikeable/Likeable, Unpleasant/Pleasant) on a five-point scale.

#### Reasons for preferences

After indicating their preferences, participants were shown the thumbnail and title for their most preferred choice and asked to describe *why they would like* to watch the video based on the thumbnail and then based on the title. Then, participants were shown the thumbnail and title for their least preferred choice and asked to describe *why they would not like* to watch the video based on the thumbnail and based on the title.

#### Thumbnail and title preference and identity matching

Participants were shown again the 12 *Deep Look* YouTube video thumbnail and titles in random order along with their four reported central identities. Participants were asked to select two thumbnails and titles that best matched their identities and two that least matched their identities. Next, participants were shown their two most preferred thumbnails and titles and their four reported central identities and then asked to explain why each image and each title was a good match with their identities. Participants were then shown their two least preferred thumbnails and titles and their four reported central identities and asked to explain why each image and each title was a mismatch with their identities.

#### Science identity

After answering questions about the thumbnails and titles, participants’ science identity was assessed using a five-point Likert scale from strongly disagree (one) to strongly agree (5) for six statements related to their science identity [62, 63]. While this scale has been used to assess STEM students’ identity rather than non-experts, the scale has been validated and review of scale items indicates appropriateness for assessing non-experts’ science identity. For example, the scale included the following statements: “Being a scientist is an important reflection of who I am,” “I have come to think of myself as a scientist.” “In general, being a scientist is an important part of my self-image,” “I feel like I belong in the field of science,” The reliability of the scale was measured using Cronbach’s alpha and achieved sufficient reliability, *α* = .95. Scores were positively skewed (floor effect, skew = 1; *M* = 1.92 of 5, *SD* = 1.04).

#### Science curiosity

Participants’ science curiosity was assessed using the Science Curiosity Scale [17, 64] that asked how closely they followed news related to various topics, how often they had conversations about these same topics, and whether they had read a book or listened to an audiobook on those topics in the previous year; topics included crime, religion, local weather, science fiction, and wildlife, among others. Consistent with prior work, the scale was evaluated and scored using item response theory (GRM Model, [65]). Scores were approximately normally distributed with a slight negative skew (skew = -0.16) and ranged from -2.12 to 2.64 (*M* = 0, *SD* = 0.94).

#### Intent to watch any video

At the end of the survey, participants were asked whether they would like to watch **any** of the videos for the thumbnails and titles featured in the survey (yes or no). This item was followed by an open-ended question asking them to explain why or why not.

#### Demographics

Demographic questions were added to the beginning of the survey to screen participants and for reporting participant characteristics. Participants were asked to self-report their age (*M* = 57.81, SD = 16.86), gender (only those who selected “woman” were able to continue the survey), race (12.18% Black, 5.93% Asian or Asian American, 70.10% White), education (37% obtained at least a college degree), annual household income (51% less than 50K, 29% between 50K and 100K, and 14% greater than 100K), voting preferences (47% typically vote for the Democratic candidate, 35% typically vote for the Republican candidate, and 18% either vote for a third-party or independent candidate, don’t typically vote, or didn’t answer the question), and political philosophy (29% liberal or very liberal, 33% moderate, 34% conservative or very conservative, and 4% didn’t answer).

*Natural language processing*. We conducted natural language processing (NLP), using procedures reported in prior research on YouTube videos [66], to identify patterns in participants’ responses to multiple, open-ended survey questions. See S3 Appendix. Term frequency was assessed because the frequency of words and phrases was an important step in understanding patterns in the data. Three lexicons (lists of words and phrases) were created from a random selection of participant responses using an iterative process, grouping related words and phrases by terms. Next, searches of related words and phrases were conducted. (For example, for the “Identity Lexicon,” some respondents mentioned race, subsequently, all other races were searched in the dataset and added to this lexicon). Derivatives of word roots and idioms were also added. Finally, similar terms were combined to represent one construct. Term frequency was calculated using C++ programming language for each individual, open-ended survey response. While the lexicons do not present all possible words and phrases associated with each term, they list frequently occurring words and phrases repeated in survey participants’ actual open-ended responses. This approach provided a more accurate assessment of responses while minimizing researcher bias.

## Results

### Finding 1: Women generally preferred the kitten and butterfly episode thumbnails and titles, stating they preferred videos that are “interesting” and “pleasant”

Our first set of research questions asked about women’s preferences for the 12 thumbnails and titles based on their selection, ranking, and rating of those thumbnails and titles. First, the “Kitten” (61%) and “Butterfly” (61%) episode thumbnails and titles were those most frequently selected by participants. In contrast, “Spider” (8%) and “Hairworm” (7%) were the least frequently selected. Second, the kitten and butterfly episodes also had the highest rankings, and the two lowest ranked episodes were the lice and hairworm videos. See Table 1. Third, participants ratings of their most and least preferred videos along the 10 bipolar adjective scales suggested that women preferred videos that they felt were "interesting," "pleasant," or "colorful." Women did not prefer videos that they felt were "disgusting," "unpleasant," and "ugly."

**Table 1:** Measures of central tendency for each episode’s ranking scores. Higher ranking videos have ranks closer to 1 and lower ranking videos have ranks closer to 12. In this table, the videos are sorted by their median ranking. Percent of viewers that are women from YouTube data are also shown.

Video Title (percent of viewers on YouTube that are women)	Median Rank	Mean (SD)	Mode (% of Responses)
How **kittens** go from clueless to cute (26%)	2	3.53 (3.23)	1 (41%)
What gives the morpho **butterfly** its magnificent blue? (18%)	2	3.51 (2.87)	1 (27%)
This vibrating **bumblebee** unlocks a flower’s hidden treasure (20%)	4	5.08 (2.99)	3 (18%)
What happens when you zap **coral** with the world’s most powerful x-ray? (11%)	5	5.61 (3.04)	3 (13%)
Why is the very hungry **caterpillar** so dang hungry? (26%)	6	6.05 (2.80)	5 (15%)
How **mosquitos** use six needles to suck your blood (21%)	7	7.07 (3.13)	9 (12%)
These whispering, walking **bats** are on to something (17%)	7	7.22 (3.16)	9 (11%)
These **fish** are all about sex on the beach (46%)	7	7.01 (3.05)	6, 8 (12%, 12%)
The snail-smashing, fish-spearing, eye-popping mantis **shrimp** (10%)	8	7.54 (2.81)	8 (13%)
For these tiny **spiders**, it’s sing or get served (28%)	8	8.05 (2.87)	12 (13%)
These **hairworms** eat a cricket alive and control its mind (21%)	9	8.59 (2.93)	11 (18%)
How **lice** turn your hair into their jungle gym (57%)	10	8.71 (3.15)	12 (23%)

These results are consistent with the NLP findings. The NLP analysis found that participants were most likely to describe their *most preferred thumbnails* using words or phrases like attractive/colorful, cute, or interesting/curious. Participants were most likely to describe their *most preferred titles* as interesting, sparking their curiosity, and being informative. Overall, the top four terms mentioned as reasons for participants’ *most preferred thumbnails and titles* were: attractive/colorful, interesting/curiosity, cute, and informative. Participants were most likely to describe their *least preferred thumbnails* using words or phrases like disgusting or by stating that they did not like that animal or wildlife shown. Participants were most likely to describe their *least preferred titles* using words or phrases like disgusting and uninteresting. Overall, the top four terms participants mentioned as reasons for their *least preferred thumbnails and titles* were: disgusting, uninteresting, don’t like the animal or wildlife, and fearful (See S2 Appendix).

It is worth noting that the kitten episode, though the most popular one in this study, was one of the least viewed on the *Deep Look* channel at the start of this project and was not popular among women: YouTube reported that only 26% of the 214,542 total viewers of the episode were women. Similarly, YouTube reported that only 18% of the 371,616 total viewers of the butterfly episode were women. Of the 12 episodes featured in this study, the lice episode was the most popular among women on the *Deep Look* channel: YouTube reported that 57% of the lice episode’s 4,147,989 total viewers were women. See Table 1. We explore the individual differences in audience preferences that help to explain this finding in finding 3 below.

### Finding 2: Few women anticipate engaging with any of the episodes

At the end of the survey, participants were asked whether they would like to view *any of the videos* featured in the survey and only 39% (n = 754 participants) said yes (61%, or 1,185 participants, said no, and 1 did not answer). This lack of interest is, perhaps, not surprising given the lack of engagement and the gender gap seen in the YouTube metrics and prior research [7]. Natural language processing findings revealed that participants who said “yes” most frequently cited expecting the videos to be interesting/curious (n = 777 mentions), informative (n = 106 mentions), and attractive/colorful (n = 62 mentions). Participants who said “no” most frequently reported that they expected the videos to be uninteresting (n = 58 mentions) and disgusting/gross (n = 37 mentions). Finding 3 discusses potential individual differences in women’s (lack of) anticipated engagement with the videos.

### Finding 3: Science curiosity predicts anticipated engagement with the episodes, generally, and science identity predicts anticipated engagement with (and higher ranking of) the less popular videos

Logistic regression analyses were conducted to examine the role of science curiosity and science identity for the “watch any episode” item and for the two most frequently selected and two least frequently selected videos. Then multiple regression analyses were conducted to examine the role of these variables in predicting the preference rankings of the top two and bottom two ranked episodes.

First, we found that both science curiosity and science identity predicted participants saying “yes” when asked whether they would want to watch any video, but science curiosity was the stronger of the two predictors. When holding science identity constant, the odds participants said “yes” increased by 90% for each unit increase in science curiosity (*b* = 0.64, *p* < .001, exp(*b*) = 1.90). In contrast, when holding science curiosity constant, the odds of saying “yes” increased by 17% for each unit increase in science identity score (*b* = 0.16, *p* = .002, exp(*b*) = 1.17).

**Fig 2:**
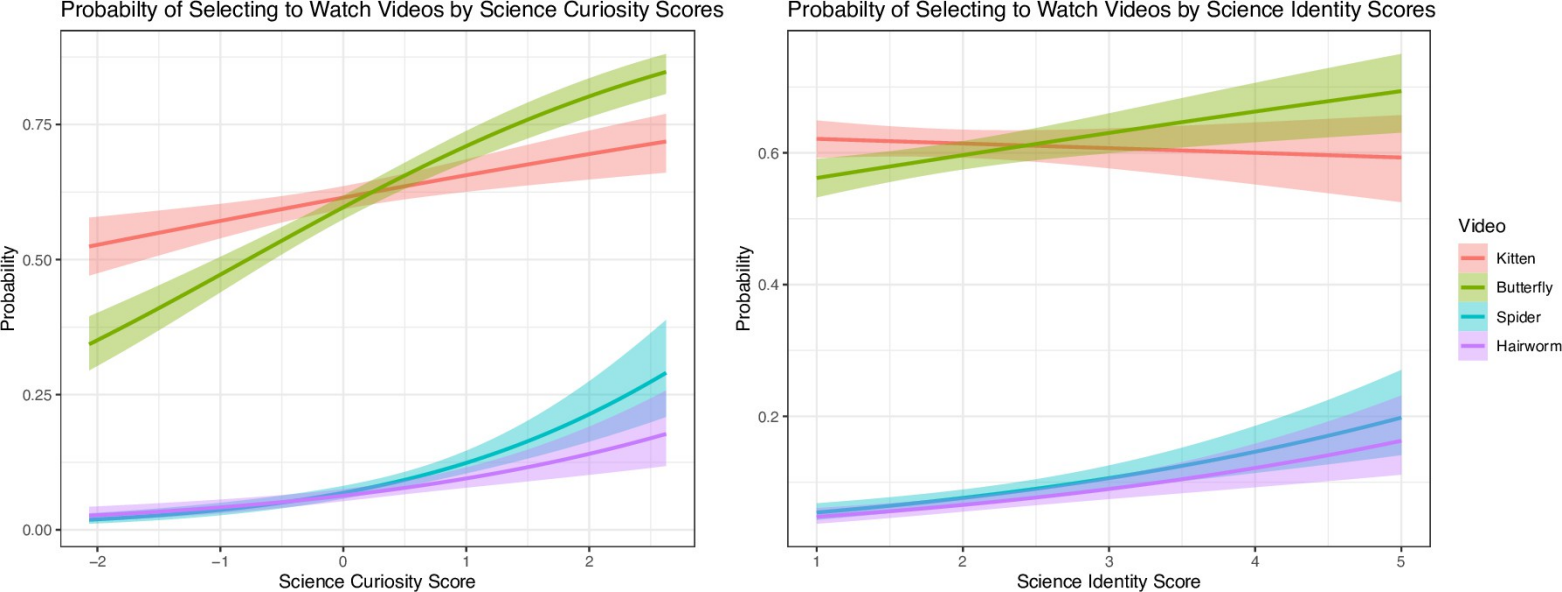
Probability of selecting to watch the kitten, butterfly, spider, and hairworm videos conditional on science curiosity (left) and science identity (right).

Next, we predicted selecting the two most frequently and two least frequently selected episodes. Holding science identity constant, for each unit increase in science curiosity, the odds of participants selecting the kitten episode increased by 25% (*b* = 0.22, exp(*b*) = 1.25, *p* < .001), the odds of participants selecting the butterfly episode increased by 68% (*b* = 0.49, exp(*b*) = 1.68, *p* < .001), the odds of selecting the spider episode increased by 78% (*b* = 0.58, exp(*b*) = 1.78, *p* < .001), and the odds of selecting the hairworm episode increased by 32% (*b* = 0.31, exp(*b*) = 1.36, *p* = .006). When holding science curiosity constant, for each unit increase in science identity, the odds of selecting the unpopular hairworm episode increased by 23% (*b* = 0.21, exp(*b*) = 1.23, *p* = .020). The odds of selecting the spider episode increased by 15%, but this effect did not meet the threshold for statistical significance (*b* = 0.14, exp(*b*) = 1.15, *p* = .099), and the odds of selecting the butterfly episode did not change (*b* = -0.04, exp(*b*) = 0.96, *p* = .444). Interestingly, holding science curiosity constant, for each unit increase in science identity score, the odds of selecting the kitten video decreased by 10%, *b* = -0.10, exp(*b*) = 0.90, *p* = .038. See Fig 2.

To examine whether participants’ rankings for these videos were conditional on science curiosity and science identity scores, each ranking was multiplied by -1 so that rankings closer to 1 (-1) were greater in value than the lower rankings (e.g., -12).

**Fig 3:**
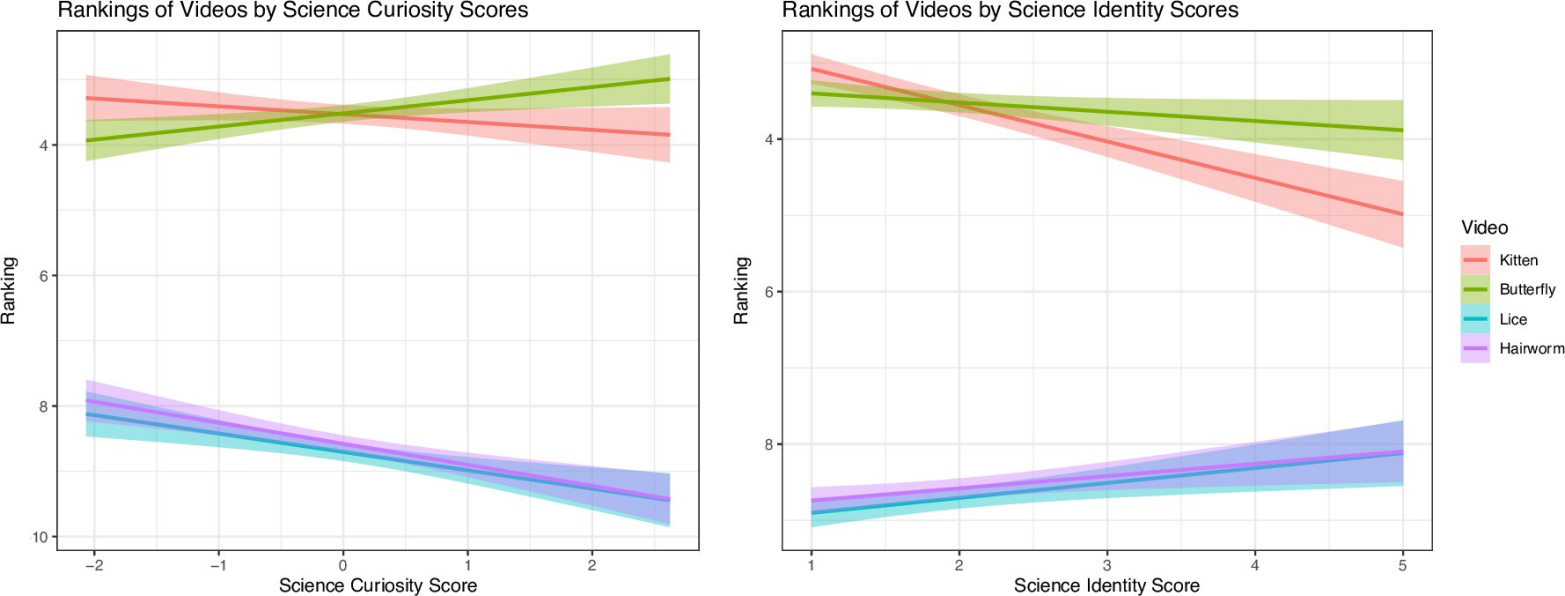
Rankings (preference scores) for the kitten, butterfly, spider, and hairworm videos conditional on science curiosity (left) and science identity (right). Better rankings (greater preferences) have ranking scores closer to 1 and worse rankings (lesser preferences) have scores closer to 12. Note that we multiplied the rankings by -1 so that scores closer to 1 (-1) are greater in value than scores with lower rankings (e.g., closer to 12, -12). To avoid confusion, the negative symbols were removed from the y-axis of the figures.

We found that greater science curiosity is associated with higher rankings of the butterfly episode (*b* = 0.26, *p* < .001, r = 0.06), lower rankings of the lice (*b* = -0.45, *p* < .001, *r* = -0.09) and hairworms episodes (*b* = -0.49, *p* < .001, *r* = -0.11), and there was no significant relationship with the kitten episode (*b* = 0.10, *p* = .237, r = -0.03). Greater science identity score, on the other hand, is associated with lower rankings for the kitten (*b* = -0.49, *p* < .001, *r* = -0.15) and butterfly (*b* = -0.19, *p* < .001, *r* = -0.05) episodes and higher rankings of the lice (*b* = 0.35, *p* < .001, *r* = 0.06) and hairworm (*b* = 0.33, *p* < .001, *r* = 0.05) episodes. See Fig 3.

### Finding 4. Women’s most central identities tended to be those describing their relationships to others and ‘colorful’, ‘attractive’, and ‘interesting’ were the descriptors of the episodes seen as most compatible with their identities

Our second set of research questions focused on which identities women reported as their most *central identities*. Identities were assessed in order to compare women’s preferences for thumbnails and titles with their most central identities identified by NLP. Women’s most central identities tended to be those describing their relationships to others: mother/grandmother (n = 797 mentions), friend (n = 679 mentions), and spouse/partner (n = 375 mentions). This is consistent with other research [67]. See Table 2.

**Table 2:** Term frequency for most central identities. This measures the number of mentions as one of the top four identities and as one of the top two identities.

Identity	Top Four Identities	Top Two Identities
Maternal (Mother/Grandmother)	797	338
Friend	679	212
Spouse/Partner	375	163
Professional	330	94
Woman	290	145
Sister	253	61
Daughter	177	46
Religious/Spiritual	109	48
Race/Ethnicity	97	26
Human	85	26
Aunt	50	10
Political affiliation	43	23
Nature/wildlife enthusiast	35	12
Sexual orientation	10	2
Generational	10	4

Findings for videos that were most compatible with participants’ top four reported identities are presented in Table 3. Participants were most likely to describe videos to be good matches with their self-reported central identities when they perceived the thumbnail or titles to be colorful (1,137), attractive (682) and/or informative (642). Findings for videos that were least compatible with their top four reported identities are presented in Table 4. Participants were most likely to describe videos to be bad matches with their self-reported central identities for videos with thumbnails and titles that were perceived to be disgusting (1,136) and/or fearful (273).

**Table 3:** Term frequency for reasons for good match between most preferred videos (thumbnails and titles) and identities.

Term	Totals: Thumbnail + Title	Thumbnails	Titles
Colorful	1,137	708	429
Attractive	682	453	229
Informative	642	235	407
Cute	632	316	316
Like/love*	608	369	239
Interesting	488	255	233
Curious	210	87	123
Unique	120	57	63
Joyful	74	38	36
Amazing	40	19	21
Humorous	27	11	16

*Statements typically referred to liking or loving the featured animal or wildlife specimen (i.e. “I love kittens,” “I like butterflies”)

**Table 4:** Term frequency for reasons for mismatch between least preferred videos (thumbnails and titles) and identities.

Term	Totals– Thumbnail + Titles	Thumbnails	Titles
Disgusting	1136	730	406
Fearful	273	155	118
Unattractive	234	180	54
Don’t like/love*	206	125	81
Uninteresting	188	73	115
Violent	78	34	44
Boring	65	39	26
Confusing	45	19	26
Irrelevant	35	19	15
Unfamiliar	34	19	15
Disease-carrying	16	8	8
Already know	8	2	6

*Statements typically referred to not liking or disliking the featured animal or wildlife specimen (i.e. “I do not like bees,” “I hate spiders”)

## Study 1 Discussion

In this first study, women’s engagement with online science media promotions, i.e., episode titles and thumbnails, and their alignment with women’s central identities were investigated. The results revealed that women generally preferred visually attractive and colorful images and topics (e.g., kittens and butterflies) over those considered disgusting or gross (e.g., hairworms, spiders, and lice) in *Deep Look* YouTube science and nature videos. However, these preferences were influenced by participants’ levels of science curiosity and science identity. Notably, women with a stronger science identity were less inclined to prefer the kitten and butterfly episodes compared to those with weaker science identity, even though they still favored these episodes overall. Additionally, science curiosity positively predicted the intention to view science content (consistent with [17] and [7]), but not all results supported this relationship. Specifically, science curiosity was not associated with selecting the kitten episode, possibly due to the high number of participants ranking the kitten video as their top choice thereby lessening the variance explainable by science curiosity. It is also worth noting the discrepancy between the kitten episode being the most popular episode amongst our particpants and the least popular episode amongst the existing *Deep Look* audience (according to the YouTube metrics), which could be seen as a limitation to our study design. One explanation for this is that the women who are engaged with *Deep Look* videos (and are not a missing audience) are a unique subsample of women who are not representative of all women; they are likely higher in science curiosity and science identity. The women in this study, however, are more diverse than those who are already engaging with *Deep Look*. Furthermore, in prior work [17], science curiosity did not predict engagement with less scientific and more entertainment-related topics. With this in mind, it is also possible that the kitten episode reads to audiences as less science-relevant. Like science curiosity, science identity also played a role, with stronger science identity associated with less desire to watch the popular kitten episode and higher rankings for the “disgusting” spider and hairworm episodes. This study suggests the importance of considering science identity in the design and content of online science media. We investigate science identity and women’s central identities in-depth in the next study.

## Study 2: Semi-structured Interviews

### Method

#### Participants

Participants were invited to volunteer for interviews by providing their email after completing the online survey (study 1), in other words, only participants recruited for study 1 were eligible to participate in study 2. To ensure diversity within the sample, a stratified sample of 24 participants was selected from a pool of 491 volunteers (25% of the survey respondents) with respect to their science curiosity scores [17] and representation in STEM by race/ethnicity as specified in national data from the National Center for Science and Engineering Statistics. Participants were first separated out by science curiosity quartiles: curious (high); open (medium-high); indifferent (low-medium); uninterested (low). Six participants from each of the four quartiles were selected to participate. Next, to ensure diverse representation, participants were selected by representation in STEM with respect to their race/ethnicity: high (Asian, Asian-American, White) or low (Black, Hispanic or Latino, Native American or Alaska Native, Native Hawaiian or Pacific Islander, or other race/ethnicity). For the six participants from each science curiosity quartile, three individuals were of white, Asian, or Asian-American race/ethnicity and three were of Black, Hispanic or Latino, Native American or Alaska Native, Native Hawaiian or Pacific Islander, or other race/ethnicity. Because of time restraints, contact with low representation in STEM interview participants was initiated before survey data had been completed. A random number generator (random.org) was used to select the order in which participants were contacted until quotas were filled. Participants’ identities reported on the survey were checked to ensure appropriate answers (e.g. nouns), and participants who had not provided appropriate identity answers were removed from consideration. A total of 146 participants were contacted for interviews between June 1 and July 7, 2021, and 24 interview participants followed-up by email and were selected. Participants were asked to provide written consent by email.

#### Procedures

Semi-structured interviews were conducted online by the second author using an interview protocol following training provided by the first author who developed the protocol (see S4 Appendix). Participants provided written consent prior to the interviews, and before recording, participants were asked to give verbal consent, keep their cameras turned off, and not mention their names or other identifying information. The decision to ask participants to keep their cameras off was reflective of the desire to allow participants as much confidentiality as possible, and no research questions were asked that would have required participants to keep their cameras on. Interviews were audio-recorded by WebEx, and transcripts provided by WebEx were downloaded, prepared, and archived for analysis. Interview transcripts were reviewed to correct minor errors and remove any inadvertent references to identifying information. Interview participants were sent a $25 gift card after the interview.

A deductive, phronetic, iterative, qualitative approach [68] was used to code themes identified in interview transcripts related to (1) women’s perceptions of science identity compatibility and (2) how women’s science identity incompatibility was linked to their identities and influenced their science engagement. Codes, words, or phrases to describe segments of qualitative data [68, 69], were identified by the first author to address the research questions. Codes were selected based on review of related theory, research, and survey data findings [24, 49]. The codes selected were science identity compatibility and science identity incompatibility; analysis of other codes that did not pertain to the focus of the current study are available online (see [70]). The second author was trained by the first author on the coding process. Both discussed examples of each code, examining examples from the dataset prior to coding, and discussed and resolved discrepancies.

The first and second authors each coded half of the interview transcripts independently, identifying data, excerpts of text embedded in the interview data, related to the codes. Following guidelines for a phronetic qualitative approach [68], interview transcripts were coded in two phases. The first phase involved primary cycle coding, assigning codes by examining participants’ responses to the interview questions [68]. The second phase involved secondary cycle coding, assigning codes to organize, synthesize, and categorizing first-level codes to explore participants’ responses [68]. Coding sheets were created by each coder, compiling data by code. Throughout the coding process, the coders met to discuss and resolve discrepancies.

## Results

### Finding 5: Most interview participants considered themselves to have a science identity or to be someone interested in science

The majority of interview participants, 16 out of 24, considered themselves to have a science identity or to be someone who is interested in science.

I am really interested about astronomy, psychology, and maybe biology. It makes me wonder. It makes me curious and, I like to read interesting stuff about those topics. *(Science Curious; Central Identities: Woman, Mother, Wife, Daughter)*

Only five interview participants did not consider themselves to have any science identity. These interview participants were from across the four groups, but only two were from the “Science Curious” group. Three interview participants described themselves as being interested in science but did not think of themselves specifically as “a science person” *(Science Indifferent; Central Identities*: *Black*, *Woman*, *African-American*, *Writer)* or as “science-oriented academically” *(Science Indifferent; Central Identities*: *Afro-American*, *Woman*, *Wife*, *Singer)*. These findings are interesting to consider further because of the overall low Science Identity scores reported for survey participants.

Interview participants who reported a science identity cited various reasons for their interest in science. Several interview participants pointed to relational watching of science television programming with spouses.

We watch documentaries in our house. My husband and I (*Science Open; Central Identities: Writer, Friend, Christian, Creator)*

We’ve always loved documentaries, wildlife documentaries, jungle animals, big cats, dolphins, you know, under-the-sea kind of documentaries. We’ll watch a lot of those. *(Science Indifferent; Central Identities*: *Christian*, *Wife*, *Mother*, *Grandmother)*

Interview participants also described an interest in collaborative science activities and projects with children. One interview participant described the importance of science on YouTube for teaching young children about science.

I have younger kids, so I’m kind of trying to get them into doing different things. Just the other day …, what was it, the Alka Seltzer in the soda bottles, and they had fun with that. They were watching YouTube videos and wanted to see how to do it themselves. Just smaller things that I’m kind of trying to get them more interested in. So different activities and things that they can do. *(Science Uninterested; Central Identities: Human, Woman, Mother, Grandmother)*

Another interview participant, although “Science Uninterested,” commented in great detail about how YouTube videos served as a teaching tool and resource for information about nature for teaching kids about science related to their personal experiences.

We kind of got into [science] because we had a bee hive, … somewhere outside of the house. And the kids are kind of scared to go outside. So, we started looking up videos on YouTube about the bees and how they make their hives, and the things they do to survive. So, they [the kids] are kind of interested in that, but it was more interesting to me, I think, just because I didn’t really know much of it. Kind of learning and explaining it to them as I’m going and listening and watching the videos too. So, things like that. There’s other [insects] like ants and things like that they’ve been finding outside. “Oh, I’m going to kill them,” or “I’m going to squish them.” I said, “No, no, no. I’m going to show you something. Here, this is how they make their homes,” and things like that. So, it’s more or less educational review as well because even though I’m sitting there with them and explaining it to them, I’m kind of giving them more of the insight as I’m watching it because I know of [some things about them]*. (Science Uninterested; Central Identities: Human, Woman, Mother, Grandmother)*

One interview participant stressed the importance of expressing a science identity because of wanting to serve as a role model for a daughter who might face racial discrimination in science.

One of the things I had to teach my children was, well, they didn’t grow up in the hood … I did … but if you have an interest in those type of subjects [i.e., science], you’re considered a nerd or you will be bullied. So, I had to kind of walk them through. My youngest daughter’s kind oflike me, she also likes science, and I had to walk her through and teach them to have pride in what they want to do and[to] just ignore people. *(Science Curious; Central Identities: Data Driven, Intelligence Seeker, Happy, content Woman, Proud Black Woman)*

Another interview participant noted an interest in science because of a personal need for health information to help a neighbor.

…. It’s just like something that’s happened the last few years… I’ve just, like, had this really deep thing about science. I look up a lot of medical stuff that is more science-related and I just enjoy it. So, that’s been [a] more recent thing for me. I had a brain injury…11 years ago… and so I’ve had ongoing issues [with sleep]… . The whys behind… why we don’t sleep? And some of it’s because I know people who have been sick, like a neighbor of ours, and some of it’s just, I don’t know, I read about something and then I get interested and I have to find out more about it. Yeah. So just very curious. I am a very curious person. (Science Open; Central Identities: Writer, Friend, Christian, Creator)

Other participants described interest in science because of an interest in the environment and as an extension of their personal observations of the natural world.

Well, a lot of it for me is environmental. I’m very big on clean energy and I actually had a friend [who] helped with a company trying to build a solar dish, a solar panel, and I actually got an MBA in environmental sciences. I just think the subject is awesome. You know, clean energy is important to me, especially living in a state like Nevada. So that’s been a strong pull for me. And actually, I’ve been doing a lot of research for a year or so about the virus, with the COVID virus, where it may come from, cures, how it might have been enhanced. That’s been so much of an interest for me. I just was actually thinking about maybe actually getting a Ph.D. in neuroscience because I find myself fascinated every day, I find myself interested in what’s going on trying to sift through the misinformation. Very interesting. I also like astrology [sic]. One of my daughters got me more into astrology. I’m fascinated by … Mars, I watched Rover go through the rocks on Mars, and NASA, NASA cam, and it’s just amazing. Well, yeah, I’m more scientifically inclined as I’ve gotten older. *(Science Curious; Central Identities: Data Driven, Intelligence Seeker, Happy, Content Woman, Proud Black Woman)*

Even several “Science Indifferent” or “Science Uninterested” interview participants described personal interest in science, which was a clear expression of science identity:

Well, I’d like to see how things how things evolve and what the reasoning is behind you know animals or plants and the climate, the earth, I’m interested in those type of things. *(Science Indifferent; Central Identities: Mother, Spouse, Grandmother, Sister)*

I have always been interested in learning. I have a higher IQ. Had advanced classes all my life in school, and I just, I love learning new things and how things work, and how they’re done, and how they’re made. New technology…. How it affects the environment. How any science affects the environment. *(Science Uninterested; Central Identities*: *Lover*, *Fighter*, *Animal Activist*, *Environmentalist)*

Various topics in science actually catch my attention, like the mosquito thing, and then now we have the cicadas here, so I’m very interested. Like, [why do] we have cicadas in the ground for so long? And they just come out. And I read [about] them and like the vaccine. (*Science Indifferent; Central Identities*: *Female*, *Asian*, *Mother*, *Engineer)*

A couple interview participants described professional connections to science, such as working as a research coordinator for clinical trials *(Science Open; Central Identities*: *Woman*, *Singer*, *Philanthropist*, *Project Manager)* and studying occupational therapy in graduate school *(Science Open; Central Identities*: *Dog [Owner]*, *Friend*, *Daughter*, *Student)*.

### Finding 6: Most participants see science engagement and having a science identity as compatible with their most central identities

The majority of interview participants (18 out of 24) considered their most central identities to be compatible with a science identity. Several interview participants noted how relational identities motivated their interest in science or nature, in general, or were linked to interest in specific *Deep Look* thumbnails and titles. Two interview participants described how interest in science was connected to their maternal identity as a mother or grandmother. For one interview participant, science engagement is seen as important for knowing how to promote good health for children.

In terms of mother, I think every mother is interested and concerned for scientific work that’s being done related to various children’s illnesses. And I know that there’s been a lot of work done by a number of the medical organizations regarding various diseases and the science related to how … various diseases affect children, and they’re working on solutions and there were trials. And so, I definitely think mother relates in that respect*. (Science Curious; Central Identities: Bird, Actress, Mother, Humorist)*

For another, science engagement is seen as important for teaching grandchildren about science.

It could be that [I’m] interested in science as a grandmother. I have grandchildren [who] ask questions. So, we have a lake place. So, they ask a lot of questions about the different things they find up at the lake. They ask about the turtles, and they ask about the fish, and so… the Grandmother part [of my identity] really relates to the science for that. (*Science Uninterested; Central Identities: Female, Mother, Grandmother, Friend)*

Even interview participants who fell into the “Science Uninterested” quartile, like the above example, described connections with science content and their identities as mothers:

It touches on that motherly instinct for me. *(Science Uninterested; Central Identities: Female, Mother, Leader, Friend)*

One participant, who was an engineer, directly discussed compatibility between her identities as a mother and as an engineer, although she did not perceive these identities to be compatible for most women:

I can be [a mother and a scientist] yes, correct, but not the general population. (*Science Indifferent; Central Identities: Female, Asian, Mother, Engineer)*

### Finding 7. Some participants believe a science identity ought to be held only by those who are working scientists

While most interview participants viewed central identities as compatible with a science identity, seven of 24 interview participants disagreed. Some distinguished between having a scientist identity and being a scientist, noting how a scientist identity and science engagement are relegated for those with an academic background in science.

In general, no, when I think when you asked [if] we [were] interested in science, I think of more of that as more of an academic kind of track and I don’t think my [identities] cover any of that. (*Science Indifferent; Central Identities: Christian, Wife, Mother, Grandmother)*

I kind of feel like they’re separate. I guess independent of the rest of it. I guess it’s just or should I say a piece of it. *(Science Open; Central Identities*: *Woman*, *Singer*, *Philanthropist*, *Project Manager)*

Interestingly, although one participant fell into the “Science Open,” quartile of science curiosity scores, this participant described an overall lack of interest in science and incompatibility with a science identity.

Anything to do with science as far as me, it’s just, I think a lack of interest in general. *(Science Open; Central Identities: Woman, Spouse, Mother, Friend)*

Some interview participants described, more generally, how social norms promote incompatibility between a science identity and other social identities, citing both sexism and racism.

Nigerian women, in particular, haven’t really been encouraged to being a scientist beyond maybe being a doctor. *(Science Uninterested; Central Identities: Friend, Woman, Nigerian, Sister)*

Women [and] womanhood have been neglected by science. I feel like the reason why science doesn’t seem appealing is because there are so many things that a woman goes through or doesn’t go through. … They’re just not catered to enough. *(Science Uninterested; Central Identities*: *Friend*, *Woman*, *Nigerian*, *Sister)*

## Study 2 Discussion

The voices and perspectives of the women featured in the interviews highlighted how science engagement is influenced by an array of factors reflective of women’s identities and science experiences. While individual differences emerged in women’s responses, as is often evident in audiences’ interpretation of media content [71, 72]; overall, women’s science engagement often was related to their relational identities—mother, grandmother, spouse, partner. This finding is consistent with work by de Oliveira et al. [16], which found that adults use YouTube to “proactively” (e.g., addressing someone’s needs) and “mutually” connect (e.g., watching YouTube together). Social identity theory [24] suggests that women associate with multiple central identities, yet certain central identities will influence their science engagement more than others. In addition, identity-based motivation theory posits that “the environment influence[s] what comes to mind when people consider who they are (the self)” [27, p. 218]. Thus, identities do, indeed, help explain why women do not engage with science media when they feel excluded.

Historically, women have been positioned as outsiders in STEM in ways that have undermined their science identities [73]. Yet, interestingly, most women interview participants, regardless of science curiosity status, described having a science identity and expressed belief that their science identity and their central identities are compatible. This finding differed from findings gathered about women’s science identity from the larger sample of women for the survey—that is, that women generally scored quite low on the measures of science identity. Future research is needed to examine the reasons for this discrepancy. One likely explanation is that the majority of the science identity scale survey items focused on professional science identity rather than everyday science identity. Regardless, women’s clear articulations of their science identity during the interviews have important implications for promoting women’s engagement with science media.

### Limitations and directions for duture research

Despite the contributions of these research findings, there are some limitations, particularly the extent to which these findings can be generalized. First, the current study only focused on videos produced by the YouTube series, *Deep Look*. This study could be replicated using other educational, digital science video series (e.g., *Physics Girl*, *It’s Okay to Be Smart*) to determine whether, and if so, to what extent the findings are generalizable. Further research with larger samples of science media products and a more in-depth analysis of product characteristics would be needed to provide more concrete advice for science communicators. Second, our survey sample did not meet national representativeness because the quota for Hispanic/Latina participants was unable to be met during the project timeframe and the average age of the sample (*M* = 57.81, *SD* = 16.9) was higher than that of the general US population (*M* = 38.9; United States Census Bureau data). That said, research could also consider oversampling from groups who are typically underrepresented in science engagement (e.g., women of color) to be able to examine intersectional differences with more statistical confidence. Additionally, while we found common themes among the 24 interview participants, these women represented around 1% of the total sample studied. Given additional resources and time, conducting additional interviews may have allowed for a more nuanced understanding of women’s preferences for science YouTube videos. Another limitation is the science identity scale has been used primarily to assess STEM students’ identity and is not a perfect fit for assessing science identity among non-experts. However, the scale has been validated, widely used, and helps assess non-expert participants’ perceptions of science identity. A new scale to capture and examine non-expert science identity could be a goal of future research.

Despite these limitations, the current study provided important foundational knowledge to better understand the role of gender, specifically, and identity, more broadly, in science engagement. Future research should consider other science topics on other YouTube videos. In addition, future research should directly compare the preferences of men and women related to their identities. Future research could also utilize eye-tracking technology to better understand attraction to science video thumbnail images and titles and influence on women’s intentions to engage. Finally, researchers should consider cross-media engagement with science videos on other social media platforms (e.g., Facebook, Instagram, TikTok) to increase engagement with younger audiences. Research on Instagram that focused on women posters who identify with STEM topics, found that often, women are not only challenging gender stereotypes, but also serving as role models for young viewers who are interested in these topics [58, 74].

## Conclusions

As science media content creators strive to be inclusive in communication design and promotion, they must consider the influence of audiences’ identities on their engagement with science media. Inclusive science communication requires science communicators to critically examine historical practices and existing assumptions about audiences in order to best serve audiences that historically have been misunderstood, marginalized, and underserved [75]. Research has identified that many potential exclusion factors, although often unconscious and unintentional, exist for audiences outside of the “typical audience for science communication” [31, p. 165]. Researchers have called for greater attention to “equity framing” in science communication as a tool for identifying alternative frames that promote science engagement by providing points of access for historically marginalized audiences and to “best frame science in ways that are salient to particular audiences” [76, p. 1].

Long-standing cultural, social, and political perspectives have reinforced traditional, masculine images of science [77]. Women are a group historically underrepresented and underserved in STEM [78]. Indeed, gender differences in science engagement are especially important to consider because of the long-standing gender gap in STEM related to women’s representation and participation in STEM [78]. Historically, gender-STEM-stereotypes have been linked to popular media portrayals of STEM professionals [79]. Yet, online science media present new opportunities for promoting more inclusive science communication for all audiences. Findings from this study highlight the need for intentional and deliberate consideration of women’s central and salient identities in online science media design to best promote inclusive science communication practices tailored to those identities. Further, this research highlighted that science curiosity and science identity are important variables that help provide additional insights when promoting science engagement for women viewers.

## Supporting information

S1 Appendix Study participant information. This appendix includes demographic information about study 1 participants and information about recruitment for study 2.
(DOCX)

S2 Appendix Preliminary analysis and scale creation. This appendix provides the code for scale creation and the results of scale evaluation. It also provides details for the analysis for study 1.
(DOCX)

S3 Appendix Notes on natural language processing. This appendix includes a note on natural language processing (NLP), the NLP lexicons, and additional term frequency tables for Study 1.
(DOCX)

S4 Appendix Semi-structured interview protocol. (DOCX)
