# Supplementary material for: Fostering inclusive science media: Insights from examining the relationship between women’s identities and their anticipated engagement with Deep Look YouTube science videos
Source: PLoS One. 2024 Aug 9;19(8):e0308558. doi: 10.1371/journal.pone.0308558 (PMC11315294; doi:10.1371/journal.pone.0308558)
Supplement: S1 Appendix — This appendix includes demographic information about study 1 participants and information about recruitment for study 2. (DOCX) [file pone.0308558.s001.docx]

**S1 Appendix. Study Participant Information.** This appendix includes demographic information about study 1 participants and information about recruitment for study 2.

## Participant Demographics from Study 1

| Variable | Sample percentage or *M* (*SD*) |
| --- | --- |
| **Age, years*** | 57.81 (16.9) |
| Generation Z (1997-2012; 18-24) | 5% |
| Millennials (1981-1996; 25-40) | 15% |
| Generation X (1965-1980; 41-56) | 16% |
| Boomers (1946-1964; 57-75) | 55% |
| Silent (1928-1945; 76-93) | 9% |
| **Race/ethnicity**** |  |
| White | 73% |
| Black or African American | 14% |
| Hispanic or Latina | 7% |
| Asian/Asian-American | 6% |
| Native American/Alaska Native | 2% |
| Native Hawaiian/Pacific Islander | 0.2% |
| Other | 2% |
| **Education** |  |
| Did not finish high school | 2% |
| High school graduate or equivalent | 24% |
| Some college, but no degree | 27% |
| Associate degree (or 2-year degree) | 10% |
| Bachelor’s degree (or 4-year degree) | 23% |
| Master’s degree | 12% |
| Doctoral degree (PhD, ED, JD, MD) | 2% |
| **Household income** |  |
| Less than $10,000 | 4% |
| $10,000 to $14,999 | 6% |
| $15,000 to $24,999 | 12% |
| $25,000 to $34,999 | 14% |
| $35,000 to $49,999 | 14% |
| $50,000 to $74,999 | 18% |
| $75,000 to $99,999 | 11% |
| $100,000 to $149,999 | 9% |
| $150,000 to $249,999 | 4% |
| $250,000 or more | 1% |
| No answer | 6% |
| **Political affiliation** |  |
| Very liberal | 12% |
| Somewhat liberal | 18% |
| Moderate | 33% |
| Somewhat conservative | 19% |
| Very conservative | 15% |
| I choose not to answer | 4% |
| **Political voting preference** |  |
| The Democratic candidate | 47% |
| The Republican candidate | 35% |
| A third-party candidate | 4% |
| I tend not to vote | 8% |
| I choose not to answer | 6% |

*Note*. *Age in years included in brackets indicates the actual age range of participants included in the study.
****Race/ethnicity category sums to more than 100% as participants were able to select more than one race/ethnicity.

## Note on Participant Recruitment for Study 2

Participants were first separated out by science curiosity quartiles: curious (high); open (medium-high); indifferent (low-medium); uninterested (low). Six participants from each of the four quartiles were selected to participate. Next to ensure diverse representation, participants were selected by representation in STEM with respect to their race/ethnicity (National Center for Science and Engineering Statistics, 2021a): high (Asian, Asian-American, White) or low (Black, Hispanic or Latino, Native American or Alaska Native, Native Hawaiian or Pacific Islander, or other race/ethnicity). For the six participants from each science curiosity quartile, three individuals were of white, Asian, or Asian-American race/ethnicity and three were of Black, Hispanic or Latino, Native American or Alaska Native, Native Hawaiian or Pacific Islander, or other race/ethnicity. Due to time restraints, contact with low representation in STEM interview participants was initiated before survey data had been completed. A random number generator (random.org) was used to select the order in which participants were contacted. Alternate interview participants were selected by the same procedure as the regular interview participants; interview participants were contacted in the order in which they responded until quotas were filled. One member of the research team examined the four identities reported in the survey to ensure appropriate answers (e.g. nouns) had been provided. Participants who had not provided appropriate identity answers were removed from consideration for interviews. A total of 146 participants were contacted for interviews.
