# Supplementary material for: Fostering inclusive science media: Insights from examining the relationship between women’s identities and their anticipated engagement with Deep Look YouTube science videos
Source: PLoS One. 2024 Aug 9;19(8):e0308558. doi: 10.1371/journal.pone.0308558 (PMC11315294; doi:10.1371/journal.pone.0308558)

# **S2 Appendix. Preliminary Analysis and Scale Creation.** This appendix provides the code for scale creation and the results of scale evaluation. It also provides details for the analysis for study 1.

# **Science Curiosity Scale Construction [15, 17, 64]**

Step 1: Recode Science Curiosity Questions

#News Interests
ni_sci = as.vector(dat$ni_sci) #scientific research or discoveries
ni_tec = as.vector(dat$ni_tech) #New technologies
ni_env = as.vector(dat$n_13) #nature or environment
ni_wild = as.vector(dat$n_14) #wildlife

#Conversations
c_sci = as.vector(dat$c_sci) #Scientific research or discoveries
c_tec = as.vector(dat$c_tech) #New Technologies
c_env = as.vector(dat$c_env) #Nature or environment
c_wild = as.vector(dat$c_13) #Wildlife

#Books
b_syfy = Recode(dat$SCS_B_3, "1=2; else=1; NA=1") #Science Fiction
b_sci = Recode(dat$SCS_B_11, "1=2; else=1; NA=1") #Science Research
b_env = Recode(dat$SCS_B_14, "1=2; else=1; NA=1") #Nature or Environment
b_wild = Recode(dat$SCS_B_15, "1=2; else=1; NA=1") #Wildlife

Step 2: Calculate SCS Score

#Combine science items into a dataframe
scs = data.frame(cbind(ni_sci, ni_tec, ni_env, ni_wild, b_syfy, b_sci, b_env, b_wild, c_sci, c_tec, c_env, c_wild))

#Run Item Response Theory model (Graded Response Model, or GRM)
grm.mod = grm(scs, constrained = F, IRT.param = T)
 plot(grm.mod)


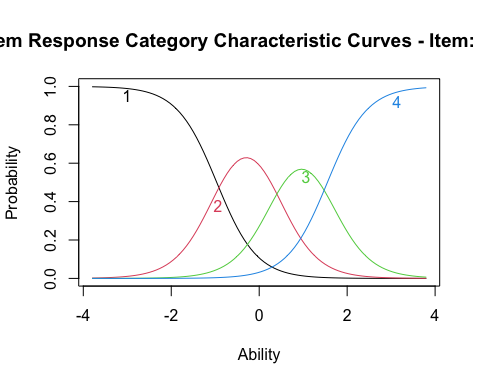


Figure S2a. Item response theory item characteristic curves - ni_sci (news interest science)


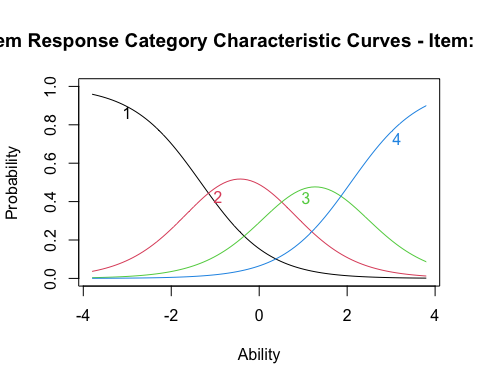


Figure S2b. Item response theory item characteristic curves – ni_tec (news interest technology)


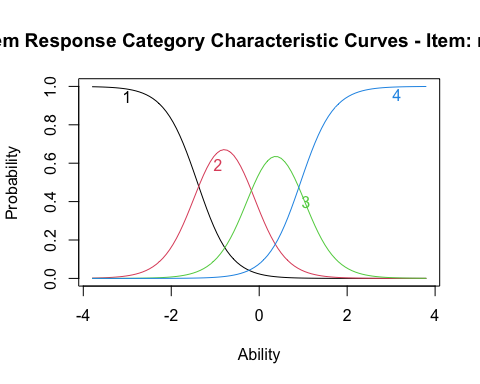


Figure S2c. Item response theory item characteristic curves – ni_env (news interest environment)


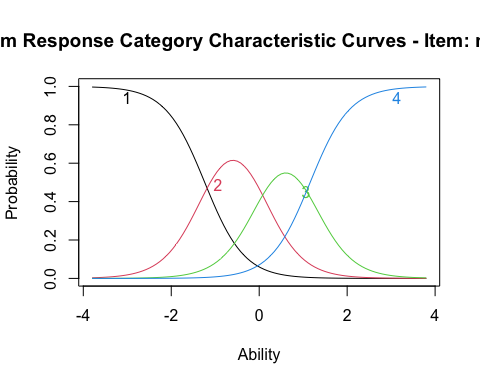


Figure S2d. Item response theory item characteristic curves – ni_wild (news interest wildlife)


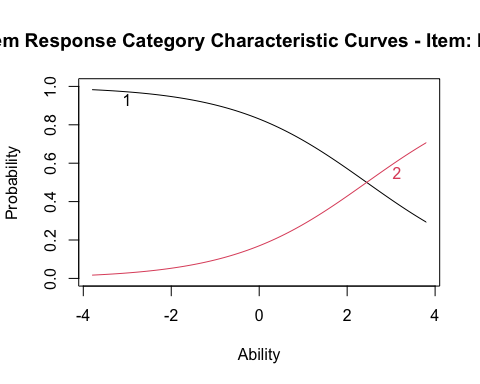


Figure S2e. Item response theory item characteristic curves – b_syfy (read a science fiction book)


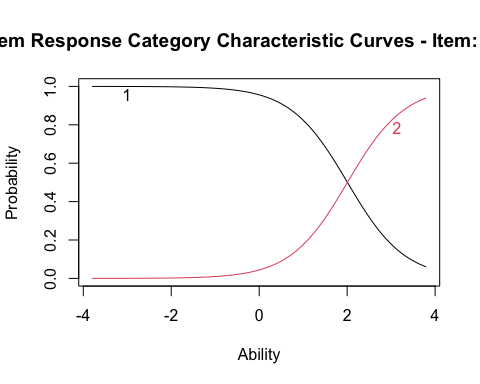


Figure S2f. Item response theory item characteristic curves – b_sci (read a book about science)


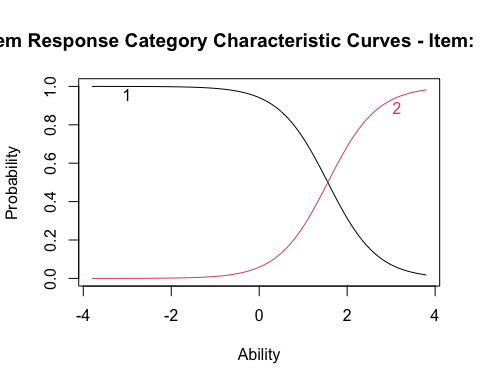


Figure S2g. Item response theory item characteristic curves – b_env (read a book about the environment)


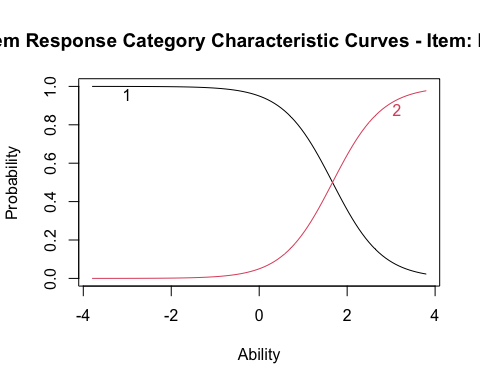


Figure S2h. Item response theory item characteristic curves – b_wild (read a book about wildlife)


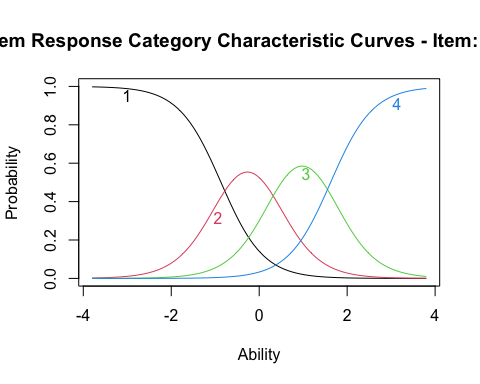


Figure S2i. Item response theory item characteristic curves – c_sci (conversations about science)


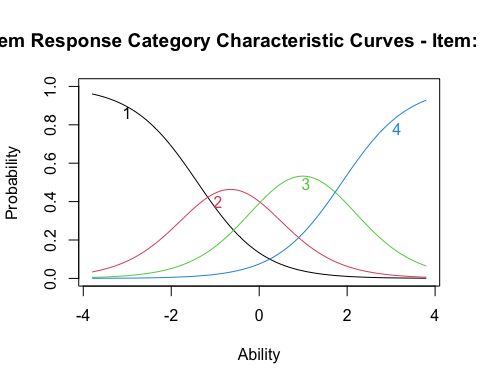


Figure S2j. Item response theory item characteristic curves – c_tec (conversations about technology)


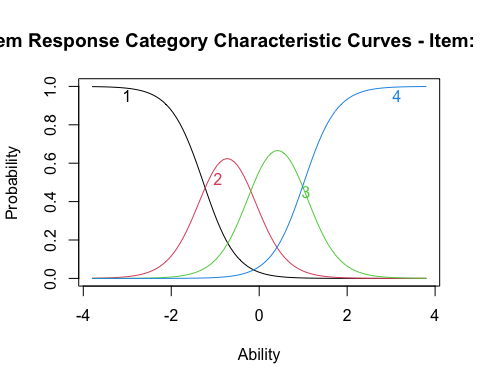


Figure S2k. Item response theory item characteristic curves – c_env (conversations about environment)


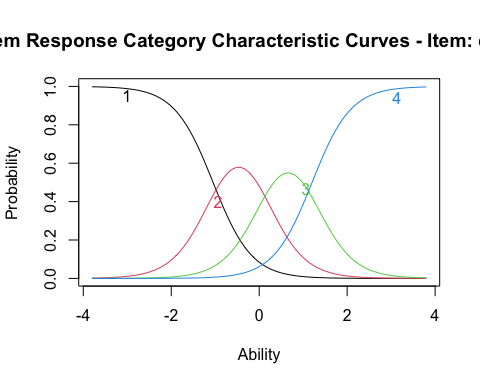


Figure S2l. Item response theory item characteristic curves – c_wild (conversations about wildlife)

Step 3: Calculate Participants’ Scores

#Calculate participants' factor scores
grm.fs = factor.scores.grm(grm.mod, resp.patterns = scs, method = "EB")
 #Extract factor scores
 dat$scs_theta = grm.fs$score.dat$z1
 dat$Zscs = as.vector(scale(dat$scs_theta, scale = T, center = T))

 sdes = as.vector(summary(dat$Zscs))
 a25 = sdes[2]
 b50 = sdes[3]
 c75 = sdes[5]

#Grouped Science Curiosity
 dat$scs_gr = rep(NA, nrow(dat))
 dat$scs_gr[dat$Zscs < a25] = 1
 dat$scs_gr[(dat$Zscs > a25 | dat$Zscs == a25) & dat$Zscs < b50] = 2
 dat$scs_gr[(dat$Zscs > b50 | dat$Zscs == b50) & dat$Zscs < c75] = 3
 dat$scs_gr[(dat$Zscs > c75 | dat$Zscs == c75)] = 4

 dat$scsGroups = factor(dat$scs_gr, levels = c(1:4), labels = c("low", "low-med", "med-high", "high"))

## Graph the Distribution of Science Curiosity Scores


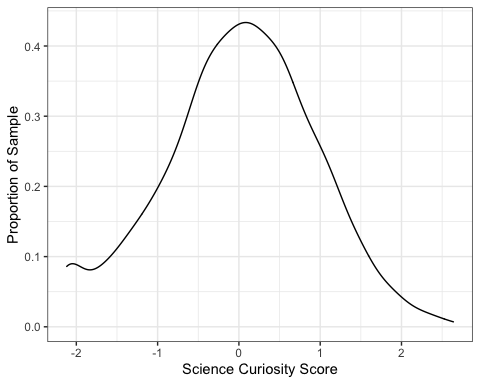


# **Science Identity Score [62, 63]**

# Calculate Science Identity Score

sid = data.frame(dat$SID_1, dat$SID_2, dat$SID_3, dat$SID_3, dat$SID_4, dat$SID_5, dat$SID_6)

psych::alpha(sid)

##
## Reliability analysis
## Call: psych::alpha(x = sid)
##
## raw_alpha std.alpha G6(smc) average_r S/N ase mean sd median_r
## 0.95 0.95 0.91 0.74 20 0.0017 1.9 1 0.74
##
## 95% confidence boundaries
## lower alpha upper
## Feldt 0.95 0.95 0.96
## Duhachek 0.95 0.95 0.96
##
## Reliability if an item is dropped:
## raw_alpha std.alpha G6(smc) average_r S/N alpha se var.r med.r
## dat.SID_1 0.95 0.95 0.89 0.75 18 0.0020 0.0074 0.74
## dat.SID_2 0.95 0.95 0.89 0.77 20 0.0018 0.0048 0.74
## dat.SID_3 0.94 0.94 0.94 0.74 17 0.0020 0.0025 0.74
## dat.SID_3.1 0.94 0.94 0.94 0.74 17 0.0020 0.0025 0.74
## dat.SID_4 0.94 0.94 0.88 0.74 17 0.0020 0.0074 0.73
## dat.SID_5 0.94 0.94 0.88 0.74 17 0.0020 0.0075 0.73
## dat.SID_6 0.95 0.95 0.89 0.75 18 0.0019 0.0071 0.74
##
## Item statistics
## n raw.r std.r r.cor r.drop mean sd
## dat.SID_1 1937 0.88 0.88 0.88 0.84 1.9 1.2
## dat.SID_2 1938 0.82 0.83 0.81 0.77 1.6 1.1
## dat.SID_3 1937 0.91 0.90 0.78 0.87 2.0 1.2
## dat.SID_3.1 1937 0.91 0.90 0.78 0.87 2.0 1.2
## dat.SID_4 1937 0.90 0.90 0.91 0.86 1.9 1.2
## dat.SID_5 1938 0.90 0.90 0.91 0.86 1.8 1.1
## dat.SID_6 1935 0.87 0.86 0.84 0.81 2.2 1.3
##
## Non missing response frequency for each item
## 1 2 3 4 5 miss
## dat.SID_1 0.53 0.17 0.16 0.09 0.04 0
## dat.SID_2 0.69 0.12 0.11 0.05 0.03 0
## dat.SID_3 0.49 0.18 0.19 0.10 0.04 0
## dat.SID_3.1 0.49 0.18 0.19 0.10 0.04 0
## dat.SID_4 0.55 0.17 0.16 0.09 0.03 0
## dat.SID_5 0.57 0.17 0.14 0.08 0.03 0
## dat.SID_6 0.45 0.18 0.16 0.15 0.06 0

dat$sid = rowMeans(sid, na.rm = T)

dat$sidgr = ifelse(dat$sid > 1.57, 1, 0)
dat$SciId = factor(dat$sidgr, levels = c(0,1), labels = c("low", "high"))

rm(sid)

## Graph the Distribution of Science Identity Scores


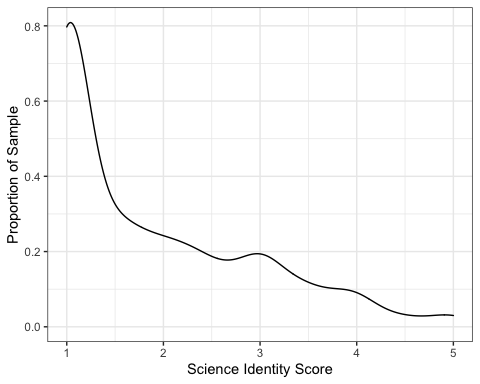


## Graph the relationship between science identity and science curiosity and examine the correlation

ggplot(data = dat, mapping = aes(x = scs_theta, y = sid)) + geom_smooth(method = "glm") + theme_bw() + labs(x = "Science Curiosity Score", y = "Science Identity Score") + coord_cartesian(ylim = c(1, 5))

## `geom_smooth()` using formula 'y ~ x'


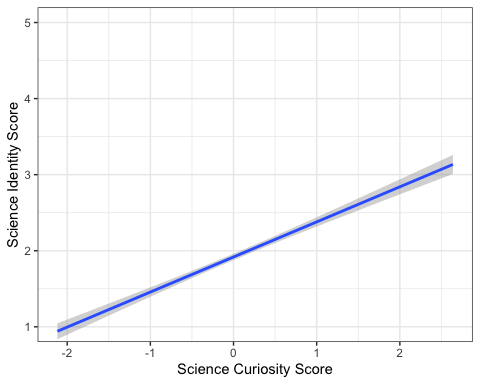


Relationship between science identity scores and science curiosity scores.

Correlation between science curiosity and science identity

cor.test(dat$scs_theta, dat$sid)

##
## Pearson's product-moment correlation
##
## data: dat$scs_theta and dat$sid
## t = 20.131, df = 1936, p-value < 2.2e-16
## alternative hypothesis: true correlation is not equal to 0
## 95 percent confidence interval:
## 0.3785384 0.4522020
## sample estimates:
## cor
## 0.4160525

# Video Selection Task

# INT Below are images and titles from YouTube videos. Please click on the image(s) of videos you would watch. You may select as many as you would like.

## Recode variables so there is a 1 if it was selected and a 0 if it was not selected.

dat$select_mosquito = Recode(dat$INT_1, "1=1; else=0; NA=0")
dat$select_butterfly = Recode(dat$INT_2, "1=1; else=0; NA=0")
dat$select_caterpillar = Recode(dat$INT_3, "1=1; else=0; NA=0")
dat$select_hairworms = Recode(dat$INT_4, "1=1; else=0; NA=0")
dat$select_bats = Recode(dat$INT_5, "1=1; else=0; NA=0")
dat$select_lice = Recode(dat$INT_6, "1=1; else=0; NA=0")
dat$select_kittens = Recode(dat$INT_7, "1=1; else=0; NA=0")
dat$select_shrimp = Recode(dat$INT_8, "1=1; else=0; NA=0")
dat$select_bee = Recode(dat$INT_9, "1=1; else=0; NA=0")
dat$select_spiders = Recode(dat$INT_10, "1=1; else=0; NA=0")
dat$select_fish = Recode(dat$INT_11, "1=1; else=0; NA=0")
dat$select_coral = Recode(dat$INT_12, "1=1; else=0; NA=0")

selected = as.vector(c(Kitten = sum(dat$select_kittens),
 Butterfly = sum(dat$select_butterfly),
 Bumblebee = sum(dat$select_bee),
 Coral = sum(dat$select_coral),
 Mosquito = sum(dat$select_mosquito),
 Caterpillar = sum(dat$select_caterpillar),
 Bat = sum(dat$select_bats),
 Fish = sum(dat$select_fish),
 Lice = sum(dat$select_lice),
 Shrimp = sum(dat$select_shrimp),
 Spider = sum(dat$select_spiders),
 Hairworm = sum(dat$select_hairworms)))

Video = c("Kitten", "Butterfly", "Bumblebee", "Coral", "Mosquito", "Caterpillar", "Bat", "Fish", "Lice", "Shrimp", "Spider", "Hairworm")

selected = data.frame(selected, Video)


colnames(selected) = c("Num_Selected", "Video")

rm(Video)

# Video Rank Task

# We multiplied each of the ranking scores by -1. We stated that we did this because 1 was the “best” ranking and 12 was the “worst” ranking. So, we thought it made more sense conceptually to have the better ranking be “greater” numerically than the worst ranking. By multiplying the values by -1, the best ranking (-1) was the highest value and the worst ranking (-12) was the lowest value.

dat$rank_mosquito = dat$RANK_1* -1
dat$rank_butterfly = dat$RANK_2 * -1
dat$rank_caterpillar = dat$RANK_3 * -1
dat$rank_hairworms = dat$RANK_4 * -1
dat$rank_bats = dat$RANK_5 * -1
dat$rank_lice = dat$RANK_6 * -1
dat$rank_kittens = dat$RANK_7 * -1
dat$rank_shrimp = dat$RANK_8 * -1
dat$rank_bee = dat$RANK_9 * -1
dat$rank_spiders = dat$RANK_10 * -1
dat$rank_fish = dat$RANK_11 * -1
dat$rank_coral = dat$RANK_12 * -1

# Video “Best” Selection

dat$bmos = Recode(dat$BMID_1, "1=1; else=0; NA=0")
dat$bbut = Recode(dat$BMID_2, "1=1; else=0; NA=0")
dat$bcat = Recode(dat$BMID_3, "1=1; else=0; NA=0")
dat$bhair = Recode(dat$BMID_4, "1=1; else=0; NA=0")
dat$bbat = Recode(dat$BMID_5, "1=1; else=0; NA=0")
dat$blice = Recode(dat$BMID_6, "1=1; else=0; NA=0")
dat$bkit = Recode(dat$BMID_7, "1=1; else=0; NA=0")
dat$bshr = Recode(dat$BMID_8, "1=1; else=0; NA=0")
dat$bbum = Recode(dat$BMID_9, "1=1; else=0; NA=0")
dat$bspi = Recode(dat$BMID_10, "1=1; else=0; NA=0")
dat$bfish = Recode(dat$BMID_11, "1=1; else=0; NA=0")
dat$bcor = Recode(dat$BMID_12, "1=1; else=0; NA=0")

# RQ1. What are women’s preferences for thumbnails and titles for Deep Look YouTube videos?

RQ1a: Which thumbnails and titles for Deep Look YouTube videos are most and least preferred by women.

RQ1b: How are these preferences associated with participants’ science curiosity and science identity scores?

RQ1c: What are the reasons for these preferences?

Participants were asked to indicate their preferences for the 12 selected Deep Look thumbnails and titles used to promote Deep Look’s YouTube videos (Figure 1). Preferences were assessed in three ways – by asking participants to **select**, **rank**, and **rate** thumbnails and titles. First, participants were randomly presented with 12 thumbnails and titles and asked to select which of the 12 videos they would watch (if any). Second, participants were asked to rank the randomly presented 12 thumbnails and titles for Deep Look videos from one to 12: one being most likely to watch and 12 being least likely to watch. Each number rank allowed for only one video to be selected for that rank. Third, for their most-liked thumbnail and title and their least-liked thumbnail and title, which were selected when ranking, participants were asked to rate these using 10 bipolar adjectives (Uninteresting/Interesting, Simple/Complex, Irrelevant/Relevant, Ugly/Beautiful, Disgusting/Charming, Not colorful/Colorful, Dull/Exciting, Unfamiliar/Familiar, Unlikeable/Likeable, Unpleasant/Pleasant) on a five-point scale.

### Select

**Which Episodes Participants Selected to Watch**

Frequency of Episode Selections

## Num_Selected Video
## 1 1203 Kitten
## 2 1174 Butterfly
## 3 704 Bumblebee
## 4 447 Coral
## 5 385 Mosquito
## 6 337 Caterpillar
## 7 304 Bat
## 8 281 Fish
## 9 164 Lice
## 10 158 Shrimp
## 11 152 Spider
## 12 130 Hairworm


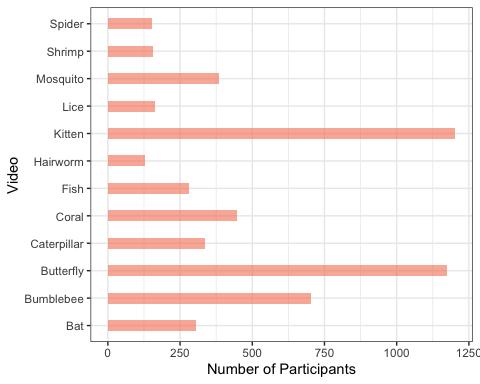


Frequencies of episode selections

**REGRESSION ANALYSES**

**Kitten Video**

##
## Call:
## glm(formula = select_kittens ~ scs_theta + sid, family = binomial(),
## data = dat)
##
## Deviance Residuals:
## Min 1Q Median 3Q Max
## -1.6137 -1.3530 0.9117 0.9846 1.2630
##
## Coefficients:
## Estimate Std. Error z value Pr(>|z|)
## (Intercept) 0.69101 0.10725 6.443 1.17e-10 ***
## scs_theta 0.22149 0.05529 4.006 6.18e-05 ***
## sid -0.10316 0.04972 -2.075 0.038 *
## ---
## Signif. codes: 0 '***' 0.001 '**' 0.01 '*' 0.05 '.' 0.1 ' ' 1
##
## (Dispersion parameter for binomial family taken to be 1)
##
## Null deviance: 2573.5 on 1937 degrees of freedom
## Residual deviance: 2557.1 on 1935 degrees of freedom
## (2 observations deleted due to missingness)
## AIC: 2563.1
##
## Number of Fisher Scoring iterations: 4

## (Intercept) scs_theta sid
## 2.00 1.25 0.90

with demographics: science identity is not significant any longer

##
## Call:
## glm(formula = select_kittens ~ scs_theta + sid + age + educ +
## black + hisp + income, family = binomial(), data = dat)
##
## Deviance Residuals:
## Min 1Q Median 3Q Max
## -1.6641 -1.3468 0.8735 0.9603 1.4775
##
## Coefficients:
## Estimate Std. Error z value Pr(>|z|)
## (Intercept) 1.0400745 0.2800053 3.714 0.000204 ***
## scs_theta 0.1962475 0.0592365 3.313 0.000923 ***
## sid -0.0642039 0.0541834 -1.185 0.236043
## age 0.0003758 0.0032243 0.117 0.907207
## educ -0.0193771 0.0379836 -0.510 0.609950
## black -0.5840373 0.1472051 -3.968 7.26e-05 ***
## hisp 0.0002414 0.2086951 0.001 0.999077
## income -0.0545116 0.0251744 -2.165 0.030361 *
## ---
## Signif. codes: 0 '***' 0.001 '**' 0.01 '*' 0.05 '.' 0.1 ' ' 1
##
## (Dispersion parameter for binomial family taken to be 1)
##
## Null deviance: 2411.7 on 1818 degrees of freedom
## Residual deviance: 2371.9 on 1811 degrees of freedom
## (121 observations deleted due to missingness)
## AIC: 2387.9
##
## Number of Fisher Scoring iterations: 4

## (Intercept) scs_theta sid age educ black
## 2.83 1.22 0.94 1.00 0.98 0.56
## hisp income
## 1.00 0.95

**Butterfly Video**

##
## Call:
## glm(formula = select_butterfly ~ scs_theta + sid, family = binomial(),
## data = dat)
##
## Deviance Residuals:
## Min 1Q Median 3Q Max
## -1.8991 -1.2721 0.8170 0.9995 1.4585
##
## Coefficients:
## Estimate Std. Error z value Pr(>|z|)
## (Intercept) 0.51696 0.10868 4.757 1.97e-06 ***
## scs_theta 0.49118 0.05727 8.577 < 2e-16 ***
## sid -0.03902 0.05095 -0.766 0.444
## ---
## Signif. codes: 0 '***' 0.001 '**' 0.01 '*' 0.05 '.' 0.1 ' ' 1
##
## (Dispersion parameter for binomial family taken to be 1)
##
## Null deviance: 2600.1 on 1937 degrees of freedom
## Residual deviance: 2513.1 on 1935 degrees of freedom
## (2 observations deleted due to missingness)
## AIC: 2519.1
##
## Number of Fisher Scoring iterations: 4

## (Intercept) scs_theta sid
## 1.68 1.63 0.96

with demographics: sid is still not significant

##
## Call:
## glm(formula = select_butterfly ~ scs_theta + sid + age + educ +
## black + hisp + income, family = binomial(), data = dat)
##
## Deviance Residuals:
## Min 1Q Median 3Q Max
## -2.0251 -1.2556 0.8017 0.9942 1.5668
##
## Coefficients:
## Estimate Std. Error z value Pr(>|z|)
## (Intercept) 0.115920 0.281219 0.412 0.68019
## scs_theta 0.492876 0.061073 8.070 7.02e-16 ***
## sid -0.012163 0.055266 -0.220 0.82581
## age 0.009404 0.003241 2.901 0.00372 **
## educ -0.031778 0.038241 -0.831 0.40598
## black 0.308534 0.153361 2.012 0.04424 *
## hisp 0.420501 0.214861 1.957 0.05034 .
## income -0.030102 0.025340 -1.188 0.23485
## ---
## Signif. codes: 0 '***' 0.001 '**' 0.01 '*' 0.05 '.' 0.1 ' ' 1
##
## (Dispersion parameter for binomial family taken to be 1)
##
## Null deviance: 2441.3 on 1818 degrees of freedom
## Residual deviance: 2346.5 on 1811 degrees of freedom
## (121 observations deleted due to missingness)
## AIC: 2362.5
##
## Number of Fisher Scoring iterations: 4

## (Intercept) scs_theta sid age educ black
## 1.12 1.64 0.99 1.01 0.97 1.36
## hisp income
## 1.52 0.97

**Spider Video**

##
## Call:
## glm(formula = select_spiders ~ scs_theta + sid, family = binomial(),
## data = dat)
##
## Deviance Residuals:
## Min 1Q Median 3Q Max
## -0.8627 -0.4409 -0.3620 -0.2843 2.8260
##
## Coefficients:
## Estimate Std. Error z value Pr(>|z|)
## (Intercept) -2.88806 0.19151 -15.080 < 2e-16 ***
## scs_theta 0.57784 0.10901 5.301 1.15e-07 ***
## sid 0.13734 0.08315 1.652 0.0986 .
## ---
## Signif. codes: 0 '***' 0.001 '**' 0.01 '*' 0.05 '.' 0.1 ' ' 1
##
## (Dispersion parameter for binomial family taken to be 1)
##
## Null deviance: 1065.6 on 1937 degrees of freedom
## Residual deviance: 1014.8 on 1935 degrees of freedom
## (2 observations deleted due to missingness)
## AIC: 1020.8
##
## Number of Fisher Scoring iterations: 5

## (Intercept) scs_theta sid
## 0.06 1.78 1.15

with demographics: sid is still marginally significant (not significant)

##
## Call:
## glm(formula = select_spiders ~ scs_theta + sid + age + educ +
## black + hisp + income, family = binomial(), data = dat)
##
## Deviance Residuals:
## Min 1Q Median 3Q Max
## -0.9792 -0.4498 -0.3603 -0.2870 2.8702
##
## Coefficients:
## Estimate Std. Error z value Pr(>|z|)
## (Intercept) -2.255717 0.464735 -4.854 1.21e-06 ***
## scs_theta 0.593041 0.111125 5.337 9.47e-08 ***
## sid 0.157003 0.088738 1.769 0.0768 .
## age -0.002538 0.005624 -0.451 0.6519
## educ -0.062914 0.067870 -0.927 0.3539
## black 0.373239 0.255320 1.462 0.1438
## hisp 0.223521 0.344945 0.648 0.5170
## income -0.063772 0.044271 -1.441 0.1497
## ---
## Signif. codes: 0 '***' 0.001 '**' 0.01 '*' 0.05 '.' 0.1 ' ' 1
##
## (Dispersion parameter for binomial family taken to be 1)
##
## Null deviance: 1031.07 on 1818 degrees of freedom
## Residual deviance: 976.88 on 1811 degrees of freedom
## (121 observations deleted due to missingness)
## AIC: 992.88
##
## Number of Fisher Scoring iterations: 5

## (Intercept) scs_theta sid age educ black
## 0.10 1.81 1.17 1.00 0.94 1.45
## hisp income
## 1.25 0.94

**Hairworms Video**

##
## Call:
## glm(formula = select_hairworms ~ scs_theta + sid, family = binomial(),
## data = dat)
##
## Deviance Residuals:
## Min 1Q Median 3Q Max
## -0.6733 -0.3968 -0.3471 -0.2985 2.6788
##
## Coefficients:
## Estimate Std. Error z value Pr(>|z|)
## (Intercept) -3.10938 0.20691 -15.028 < 2e-16 ***
## scs_theta 0.30961 0.11155 2.776 0.00551 **
## sid 0.20512 0.08848 2.318 0.02043 *
## ---
## Signif. codes: 0 '***' 0.001 '**' 0.01 '*' 0.05 '.' 0.1 ' ' 1
##
## (Dispersion parameter for binomial family taken to be 1)
##
## Null deviance: 953.57 on 1937 degrees of freedom
## Residual deviance: 930.29 on 1935 degrees of freedom
## (2 observations deleted due to missingness)
## AIC: 936.29
##
## Number of Fisher Scoring iterations: 5

## (Intercept) scs_theta sid
## 0.04 1.36 1.23

with demographics: sid is no longer significant

##
## Call:
## glm(formula = select_hairworms ~ scs_theta + sid + age + educ +
## black + hisp + income, family = binomial(), data = dat)
##
## Deviance Residuals:
## Min 1Q Median 3Q Max
## -1.0547 -0.4065 -0.3233 -0.2621 2.7022
##
## Coefficients:
## Estimate Std. Error z value Pr(>|z|)
## (Intercept) -1.245209 0.469027 -2.655 0.007934 **
## scs_theta 0.433552 0.115454 3.755 0.000173 ***
## sid 0.097722 0.095061 1.028 0.303954
## age -0.021389 0.005724 -3.737 0.000186 ***
## educ 0.020398 0.071925 0.284 0.776720
## black 0.429824 0.253371 1.696 0.089806 .
## hisp 0.192793 0.338752 0.569 0.569270
## income -0.125128 0.047020 -2.661 0.007787 **
## ---
## Signif. codes: 0 '***' 0.001 '**' 0.01 '*' 0.05 '.' 0.1 ' ' 1
##
## (Dispersion parameter for binomial family taken to be 1)
##
## Null deviance: 915.84 on 1818 degrees of freedom
## Residual deviance: 861.80 on 1811 degrees of freedom
## (121 observations deleted due to missingness)
## AIC: 877.8
##
## Number of Fisher Scoring iterations: 6

## (Intercept) scs_theta sid age educ black
## 0.29 1.54 1.10 0.98 1.02 1.54
## hisp income
## 1.21 0.88

## `geom_smooth()` using formula 'y ~ x'


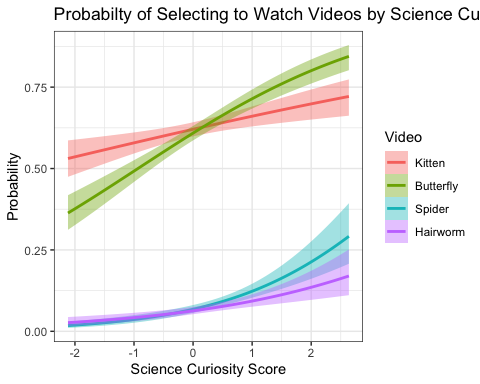


Figure S6. Probabilities of selecting to watch each episode by science curiosity score.

## `geom_smooth()` using formula 'y ~ x'


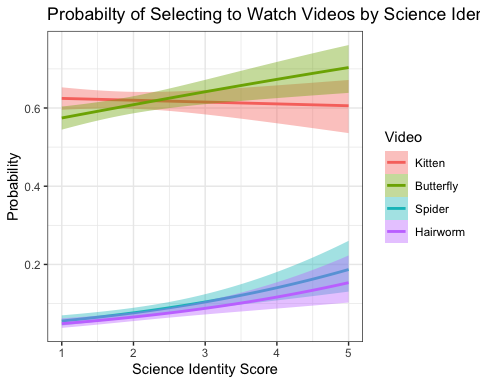


Figure S7. Probabilites of selecting to watch each episode by science identity score.

### Rank

**Kitten Video**

##
## Call:
## glm(formula = rank_kittens ~ scs_theta + sid, data = dat)
##
## Deviance Residuals:
## Min 1Q Median 3Q Max
## -9.087 -1.446 1.266 2.168 4.110
##
## Coefficients:
## Estimate Std. Error t value Pr(>|t|)
## (Intercept) -2.52686 0.16507 -15.308 < 2e-16 ***
## scs_theta 0.10162 0.08589 1.183 0.237
## sid -0.49368 0.07733 -6.384 2.17e-10 ***
## ---
## Signif. codes: 0 '***' 0.001 '**' 0.01 '*' 0.05 '.' 0.1 ' ' 1
##
## (Dispersion parameter for gaussian family taken to be 9.941519)
##
## Null deviance: 19013 on 1871 degrees of freedom
## Residual deviance: 18581 on 1869 degrees of freedom
## (68 observations deleted due to missingness)
## AIC: 9617
##
## Number of Fisher Scoring iterations: 2

with demographics: significant results don’t change, scs is still not significant

##
## Call:
## glm(formula = rank_kittens ~ scs_theta + sid + age + educ + black +
## hisp + income, data = dat)
##
## Deviance Residuals:
## Min 1Q Median 3Q Max
## -9.514 -1.417 1.280 2.110 4.712
##
## Coefficients:
## Estimate Std. Error t value Pr(>|t|)
## (Intercept) -3.36392 0.42762 -7.867 6.32e-15 ***
## scs_theta -0.02844 0.09123 -0.312 0.755324
## sid -0.36261 0.08368 -4.333 1.55e-05 ***
## age 0.01791 0.00495 3.618 0.000306 ***
## educ -0.04914 0.05862 -0.838 0.401960
## black -1.01785 0.23215 -4.384 1.23e-05 ***
## hisp -0.27724 0.32572 -0.851 0.394785
## income -0.02719 0.03863 -0.704 0.481614
## ---
## Signif. codes: 0 '***' 0.001 '**' 0.01 '*' 0.05 '.' 0.1 ' ' 1
##
## (Dispersion parameter for gaussian family taken to be 9.928345)
##
## Null deviance: 18334 on 1759 degrees of freedom
## Residual deviance: 17394 on 1752 degrees of freedom
## (180 observations deleted due to missingness)
## AIC: 9044.5
##
## Number of Fisher Scoring iterations: 2

**Butterfly Video**

##
## Call:
## glm(formula = rank_butterfly ~ scs_theta + sid, data = dat)
##
## Deviance Residuals:
## Min 1Q Median 3Q Max
## -8.9504 -0.8823 1.1718 2.0989 3.4722
##
## Coefficients:
## Estimate Std. Error t value Pr(>|t|)
## (Intercept) -3.06498 0.14655 -20.914 < 2e-16 ***
## scs_theta 0.26196 0.07626 3.435 0.000605 ***
## sid -0.19294 0.06866 -2.810 0.005002 **
## ---
## Signif. codes: 0 '***' 0.001 '**' 0.01 '*' 0.05 '.' 0.1 ' ' 1
##
## (Dispersion parameter for gaussian family taken to be 7.836706)
##
## Null deviance: 14757 on 1871 degrees of freedom
## Residual deviance: 14647 on 1869 degrees of freedom
## (68 observations deleted due to missingness)
## AIC: 9171.6
##
## Number of Fisher Scoring iterations: 2

with demographics: results don’t change much, both are still significant

##
## Call:
## glm(formula = rank_butterfly ~ scs_theta + sid + age + educ +
## black + hisp + income, data = dat)
##
## Deviance Residuals:
## Min 1Q Median 3Q Max
## -9.231 -1.024 1.043 2.030 4.193
##
## Coefficients:
## Estimate Std. Error t value Pr(>|t|)
## (Intercept) -4.892073 0.380353 -12.862 < 2e-16 ***
## scs_theta 0.217687 0.081150 2.683 0.00738 **
## sid -0.160258 0.074432 -2.153 0.03145 *
## age 0.022173 0.004403 5.036 5.25e-07 ***
## educ 0.088254 0.052141 1.693 0.09071 .
## black -0.108660 0.206493 -0.526 0.59880
## hisp -0.118534 0.289715 -0.409 0.68249
## income 0.028068 0.034362 0.817 0.41414
## ---
## Signif. codes: 0 '***' 0.001 '**' 0.01 '*' 0.05 '.' 0.1 ' ' 1
##
## (Dispersion parameter for gaussian family taken to be 7.854883)
##
## Null deviance: 14161 on 1759 degrees of freedom
## Residual deviance: 13762 on 1752 degrees of freedom
## (180 observations deleted due to missingness)
## AIC: 8632.2
##
## Number of Fisher Scoring iterations: 2

**Lice Video**

##
## Call:
## glm(formula = rank_lice ~ scs_theta + sid, data = dat)
##
## Deviance Residuals:
## Min 1Q Median 3Q Max
## -4.7138 -2.5321 -0.9326 1.9745 8.8426
##
## Coefficients:
## Estimate Std. Error t value Pr(>|t|)
## (Intercept) -9.44865 0.16220 -58.254 < 2e-16 ***
## scs_theta -0.44601 0.08440 -5.284 1.41e-07 ***
## sid 0.34849 0.07599 4.586 4.81e-06 ***
## ---
## Signif. codes: 0 '***' 0.001 '**' 0.01 '*' 0.05 '.' 0.1 ' ' 1
##
## (Dispersion parameter for gaussian family taken to be 9.598972)
##
## Null deviance: 18274 on 1871 degrees of freedom
## Residual deviance: 17940 on 1869 degrees of freedom
## (68 observations deleted due to missingness)
## AIC: 9551.3
##
## Number of Fisher Scoring iterations: 2

with demographics: results don’t change much, both are still significant

##
## Call:
## glm(formula = rank_lice ~ scs_theta + sid + age + educ + black +
## hisp + income, data = dat)
##
## Deviance Residuals:
## Min 1Q Median 3Q Max
## -5.2593 -2.4864 -0.7951 1.9685 9.1282
##
## Coefficients:
## Estimate Std. Error t value Pr(>|t|)
## (Intercept) -7.119886 0.414749 -17.167 < 2e-16 ***
## scs_theta -0.288459 0.088489 -3.260 0.00114 **
## sid 0.255927 0.081163 3.153 0.00164 **
## age -0.027790 0.004801 -5.788 8.43e-09 ***
## educ -0.033318 0.056857 -0.586 0.55795
## black 0.624797 0.225167 2.775 0.00558 **
## hisp 0.170828 0.315914 0.541 0.58875
## income -0.099097 0.037469 -2.645 0.00825 **
## ---
## Signif. codes: 0 '***' 0.001 '**' 0.01 '*' 0.05 '.' 0.1 ' ' 1
##
## (Dispersion parameter for gaussian family taken to be 9.339788)
##
## Null deviance: 17265 on 1759 degrees of freedom
## Residual deviance: 16363 on 1752 degrees of freedom
## (180 observations deleted due to missingness)
## AIC: 8937
##
## Number of Fisher Scoring iterations: 2

**Hairworms Video**

##
## Call:
## glm(formula = rank_hairworms ~ scs_theta + sid, data = dat)
##
## Deviance Residuals:
## Min 1Q Median 3Q Max
## -4.8103 -2.2802 -0.7046 1.9232 8.0362
##
## Coefficients:
## Estimate Std. Error t value Pr(>|t|)
## (Intercept) -9.25708 0.15096 -61.322 < 2e-16 ***
## scs_theta -0.48835 0.07855 -6.217 6.24e-10 ***
## sid 0.33293 0.07072 4.708 2.69e-06 ***
## ---
## Signif. codes: 0 '***' 0.001 '**' 0.01 '*' 0.05 '.' 0.1 ' ' 1
##
## (Dispersion parameter for gaussian family taken to be 8.314808)
##
## Null deviance: 15906 on 1871 degrees of freedom
## Residual deviance: 15540 on 1869 degrees of freedom
## (68 observations deleted due to missingness)
## AIC: 9282.5
##
## Number of Fisher Scoring iterations: 2

with demographics: results don’t change much. Both are still significant

##
## Call:
## glm(formula = rank_hairworms ~ scs_theta + sid + age + educ +
## black + hisp + income, data = dat)
##
## Deviance Residuals:
## Min 1Q Median 3Q Max
## -4.9480 -2.2673 -0.7166 1.9912 8.1396
##
## Coefficients:
## Estimate Std. Error t value Pr(>|t|)
## (Intercept) -8.201381 0.389986 -21.030 < 2e-16 ***
## scs_theta -0.405626 0.083206 -4.875 1.19e-06 ***
## sid 0.278994 0.076317 3.656 0.000264 ***
## age -0.014738 0.004515 -3.264 0.001118 **
## educ 0.005117 0.053462 0.096 0.923767
## black 0.543323 0.211723 2.566 0.010364 *
## hisp 0.369015 0.297052 1.242 0.214309
## income -0.041225 0.035232 -1.170 0.242123
## ---
## Signif. codes: 0 '***' 0.001 '**' 0.01 '*' 0.05 '.' 0.1 ' ' 1
##
## (Dispersion parameter for gaussian family taken to be 8.257805)
##
## Null deviance: 15064 on 1759 degrees of freedom
## Residual deviance: 14468 on 1752 degrees of freedom
## (180 observations deleted due to missingness)
## AIC: 8720.3
##
## Number of Fisher Scoring iterations: 2


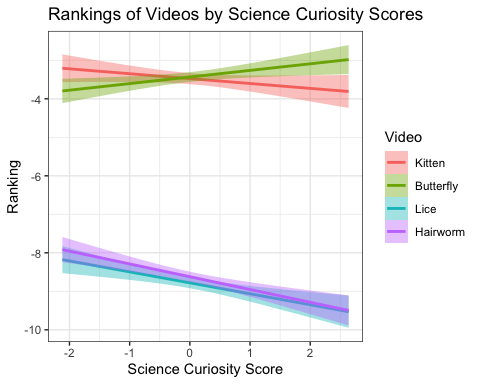


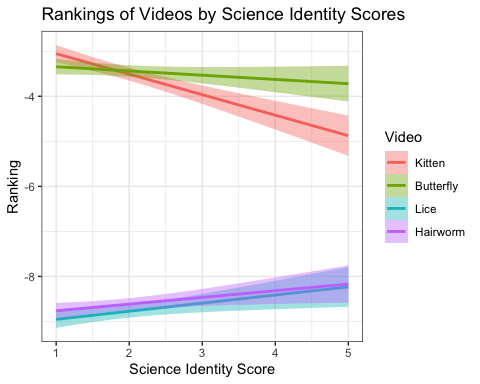

Supplement: S2 Appendix — This appendix provides the code for scale creation and the results of scale evaluation. It also provides details for the analysis for study 1. (DOCX) [file pone.0308558.s002.docx]
