# Supplementary material for: Fostering inclusive science media: Insights from examining the relationship between women’s identities and their anticipated engagement with Deep Look YouTube science videos
Source: PLoS One. 2024 Aug 9;19(8):e0308558. doi: 10.1371/journal.pone.0308558 (PMC11315294; doi:10.1371/journal.pone.0308558)
Supplement: S3 Appendix — This appendix includes a note on natural language processing (NLP), the NLP lexicons, and additional term frequency tables for Study 1. (DOCX) [file pone.0308558.s003.docx]

**S3 Appendix. Notes on Natural Language Processing.** This appendix includes a note on natural language processing (NLP), the NLP lexicons, and additional term frequency tables for Study 1.

Natural language processing [66] uses automated text analysis to identify trends and patterns from large amount of data generated from participants’ responses to open-ended survey questions.

Term frequency, or the frequency of appearance of words and phrases, was used to assess participants’ reactions to the *Deep Look*thumbnails and titles. Three lexicons were developed to address several of the research questions. Each lexicon included detailed lists of words and phrases derived from participants’ open-ended survey responses. This approach provided a more accurate assessment of participants’ responses while minimizing researcher bias. One member of the research team created the lexicons from a random selection of participant responses using an iterative process, grouping related words and phrases by term. Next, related word and phrase searches were conducted to identify related words and phrases appearing in the dataset. For example, for the “Identity Lexicon,” some respondents mentioned race, subsequently, all other races were searched in the dataset and added to the lexicon. In addition, derivatives of word roots and idioms were added to the lexicons. Finally, terms that were similar were combined to represent one construct. It is important to note that while the lexicons do not present all possible words and phrases associated with each term, they list frequently occurring, repeating patterns of words and phrases found in survey participants’ open-ended responses.

Term frequency was determined through automated text analysis. Assessing term frequency is an important step in understanding patterns from unstructured text data [66]. Excel data files for participants’ responses for each open-ended survey question were prepared for analysis. Spelling errors of words and phrases were identified and corrected prior to analysis. Words and phrases were converted to all lowercase letters to prevent uppercase/lowercase sensitivity. All responses for each open-ended question analyzed were considered as a single corpus, and term frequency analysis was conducted using C++ programming language. Each open-ended survey question response dataset was matched with one of the lexicons specifically created for this study (Appendix A). The data were read from a CSV file by using C++ programming language. Each response was processed separately and then added to the total count of words and phrases associated with each term. Word and phrases were searched by each term. A map data structure was used to store the word and phrase counts by term. Each response was stored as a string. If the word or phrase was found, it was counted and removed from the string to prevent duplicates. Term frequency counts for each participant were written to a CSV file with each column representing a term and each row representing a single participant. Totals across all participants were calculated for each open-ended survey question analyzed. These data generated from natural language processing was used to identify themes for analysis of data from the semi-structured interviews.

Reference 66: Das S, Dutta A, Lindheimer T, Jalayer M, Elgart Z. YouTube as a source of information in understanding autonomous vehicle consumers: natural language processing study. Transportation Research Record. 2019; 2673(8): 242-23. <https://doi.org/10.1177/0361198119842110>

# **Natural Language Processing Lexicons**

| *Natural Language Processing Liked Lexicon* | |
| --- | --- |
| **Term** | **Words/Word Phrases** |
| Interest* | “interest”, “interesting”, “interested”, “fascinate”, “fascinating”, “fascinated”, “engage”, “engaging”, “engaged”, “entice”, “enticing”, “enticed”, “awesome”, “fantastic” |
| Cuteness | “cute”, “cutest”, “sweet”, “sweetest”, “adorable”, “cuddly” |
| Curious* | “curious”, “curiosity”, “intrigue”, “intriguing”, “intrigued”, “want to see”, “wanna see”, “fascinate”, “fascinated”, “fascinating” |
| Informative | “informative”, “informational”, “informed”, “education”, “educational”, “know”, “learn”, “learning”, “learned”, “know”, “knew”, “educated” |
| Colorful** | “color”, “colorful”, “coloring”, “hues”, “bright”, “vibrant”, “blue”, “purple”, “green”, “yellow” |
| Unique | “unique”, “unusual”, “unusualness”, “different”, “innovative” |
| Attractive** | “attractive”, “attract”, “attracting”, “attracted”, “beautiful”, “gorgeous”, “pretty”, “prettiest”, “prettier”, “appeal”, “appealing”, “appealed” |
| Happiness | “happy”, happier,” “happiest”, “heart-warming”, “joy”, “warm” |
| Amazement* | “amaze”, “amazing”, “amazed”, “amazement”, “cool”, “coolest” |
| “Like/Love” (General) | “I like”, “I liked”, “liking”, “I love”, “I loved”, “I am loving”, “I’m loving”, “I adore,” “I adored”, “adoring” |
| *Categories combined to create “Interest/Curiosity”  ** Categories combined to create “Attractive/Colorful” | |

| *Natural Language Processing Not Liked Lexicon* | |
| --- | --- |
| **Term** | **Words/Word Phrases** |
| Boring | “bore”, “boring”, “bored”, “dull”, “do not care”, “don’t care”, “don’t really care”, “not a fan”, “not a big fan” |
| Uninteresting | “not of interest”, “not interesting”, “not interested”, “no interest”, “does not interest”, “doesn’t interest”, “not something I want to know”; “don’t want to know”, “do not want to know”, “do not need to know”, “don’t need to know”, “uninteresting”, “doesn’t sound interesting”, “does not sound interesting” |
| Unattractive | “unattractive”, “ugly”, “ugliest”, “uglier”, “not pretty”, “does not appeal”, “doesn’t appeal”, “dreary”, “didn’t find it attractive”, “not attractive” |
| Disgust | “gross”, “disgusting”, “unpleasant”, “terrible”, “graphic”, “too graphic”, “cringe”, “cringy”, “cringe-worthy”, “nasty”, “horrible”, “creeps”, “creepy”, “creeps me out”, “creeped out”, “awful”, “morbid”, “not nice”, “eww”, “gory”, “icky” |
| Fear | “scare”, “scaring”, “scared”, “scariest”, “scary”, “fright”, “frightening”, “frightened”, “fear”, “fearing”, “feared”, “fearful”, “evil”, “violent”, “horror”, “horrible”, “horrifying”, “shudder”, “shudders”, “nightmare”, “nightmares”, “arachnophobia”, “arachnophobic”, *“*ichthyophobia”, “ichthyophobic”**, “**anxious” |
| Unfamiliar | “unfamiliar”, “weird” |
| Not relevant | “not relevant”, “irrelevant”, “do not relate”, “cannot relate”, “can not relate”,  “no relation”, “not related” |
| Known | “already know”, “I know”, “knowledge”, “knowledgeable” |
| Disease | “carries germs”, “carry germs”, “carries disease”, “carry disease”, “spread germ”, “spreads germs”, “spread disease”, “spreads disease”, “ailments” |
| Confused | “confuse”, “confusing”, “confused”, “unclear”, “puzzling”, “not sure” |
| Violent/Aggressive | “violent”, “violence”, “aggressive”’ “aggression”, “conflict”, “anger”, “angry” |
| Hate/Don’t Like  (General) | “averse”, “aversion”, “I do not like” “I did not like”, “I don’t like”, “ I didn’t like” “I do not love”, “I did not love”, “I don’t love”, “I didn’t love”, “I hate” |

| *Natural Language Processing Not Liked + Time Constraints Lexicon* | |
| --- | --- |
| **Term** | **Words/Word Phrases** |
| Boring | “bore”, “boring”, “bored”, “dull”, “do not care”, “don’t care”, “don’t really care”, “not a fan”, “not a big fan” |
| Uninteresting | “not of interest”, “not interesting”, “not interested”, “no interest”, “does not interest”, “doesn’t interest”, “not something I want to know”; “don’t want to know”, “do not want to know”, “do not need to know”, “don’t need to know”, “uninteresting”, “doesn’t sound interesting”, “does not sound interesting”, “nothing really interested me” |
| Unattractive | “unattractive”, “ugly”, “ugliest”, “uglier”, “not pretty”, “does not appeal”, “doesn’t appeal”, “dreary”, “didn’t find it attractive”, “not attractive” |
| Disgust | “gross”, “disgusting”, “unpleasant”, “terrible”, “graphic”, “too graphic”, “cringe”, “cringy”, “cringe-worthy”, “nasty”, “horrible”, “creeps”, “creepy”, “creeps me out”, “creeped out”, “awful”, “morbid”, “not nice”, “eww”, “gory”, “icky” |
| Fear | “scare”, “scaring”, “scared”, “scariest”, “scary”, “fright”, “frightening”, “frightened”, “fear”, “fearing”, “feared”, “fearful”, “evil”, “violent”, “horror”, “horrible”, “horrifying”, “shudder”, “shudders”, “nightmare”, “nightmares”, “arachnophobia”, “arachnophobic”, *“*ichthyophobia”, “ichthyophobic”**, “**anxious” |
| Unfamiliar | “unfamiliar”, “weird” |
| Not relevant | “not relevant”, “irrelevant”, “do not relate”, “cannot relate”, “can not relate”,  “no relation”, “not related” |
| Known | “already know”, “I know”, “knowledge”, “knowledgeable” |
| Disease | “carries germs”, “carry germs”, “carries disease”, “carry disease”, “spread germ”, “spreads germs”, “spread disease”, “spreads disease”, “ailments” |
| Confused | “confuse”, “confusing”, “confused”, “unclear”, “puzzling”, “not sure” |
| Violent/Aggressive | “violent”, “violence”, “aggressive”’ “aggression”, “conflict”, “anger”, “angry” |
| Hate/Don’t Like  (General) | “averse”, “aversion”, “I do not like” “I did not like”, “I don’t like”, “ I didn’t like” “I do not love”, “I did not love”, “I don’t love”, “I didn’t love”, “I hate” |
| Time Constraints | “no time”, “don’t have time”, “don’t have the time”, “busy”, “too busy”, “can’t watch right now” |

## Term Frequency Tables

## *Reasons for Most Preferred Thumbnail and Title*

| **Term** | **Total:**  **Thumbnail + Title (Combined)** | **Thumbnail** | **Title** |
| --- | --- | --- | --- |
| Attractive/Colorful* | 1,068 | 676 | 392 |
| Interesting/Curious** | 791 | 393 | 398 |
| Cute | 709 | 416 | 293 |
| Informative | 503 | 164 | 339 |
| Like/love*** | 142 | 107 | 35 |
| Amazing | 40 | 19 | 21 |
| Joyful | 34 | 21 | 13 |
| Unique | 32 | 17 | 15 |
| Humorous | 32 | 13 | 19 |

*Terms combined to create one construct: “Attractive/Colorful”

**Terms combined to create one construct: “Interesting/Curious”

***Statements typically referred to liking or loving a featured animal or wildlife specimen (i.e. “I like butterflies,” “I love kittens”)

## *Reasons for Least Preferred Thumbnail and Title*

| **Term** | **Total:**  **Thumbnail + Title (Combined)** | **Thumbnail** | **Title** |
| --- | --- | --- | --- |
| Disgusting | 786 | 524 | 262 |
| Uninteresting | 216 | 127 | 89 |
| Don’t like/hate (General)* | 200 | 177 | 23 |
| Fearful | 136 | 87 | 49 |
| Boring | 89 | 51 | 38 |
| Unattractive | 83 | 70 | 13 |
| Confusing | 31 | 17 | 14 |
| Unfamiliar | 23 | 15 | 8 |
| Already know | 13 | 6 | 7 |
| Violent | 11 | 4 | 7 |
| Disease-carrying | 4 | 2 | 2 |
| Irrelevant | 5 | 1 | 4 |

*Statements typically referred to not liking or disliking the featured animal or wildlife specimen (i.e. “I do not like bees,” “I hate spiders)

## *Most Central Social Identities*

| **Identity** | **Top Four Identities** | **Top Two Identities** |
| --- | --- | --- |
| Maternal (Mother/Grandmother) | 797 | 338 |
| Friend | 679 | 212 |
| Spouse/Partner | 375 | 163 |
| Professional | 330 | 94 |
| Woman | 290 | 145 |
| Sister | 253 | 61 |
| Daughter | 177 | 46 |
| Religious/Spiritual | 109 | 48 |
| Race/Ethnicity | 97 | 26 |
| Human | 85 | 26 |
| Aunt | 50 | 10 |
| Political affiliation | 43 | 23 |
| Nature/wildlife enthusiast | 35 | 12 |
| Sexual orientation | 10 | 2 |
| Generational | 10 | 4 |
