# Supplementary material for: Fostering inclusive science media: Insights from examining the relationship between women’s identities and their anticipated engagement with Deep Look YouTube science videos
Source: PLoS One. 2024 Aug 9;19(8):e0308558. doi: 10.1371/journal.pone.0308558 (PMC11315294; doi:10.1371/journal.pone.0308558)
Supplement: S4 Appendix — (DOCX) [file pone.0308558.s004.docx]

**S4 Appendix. Semi-structured Interview Protocol.**

***Introduction and Consent***

Thank you for your time today. We would like to ask you some questions about the images and titles for some videos that you saw on the survey you completed recently.

We will be audio recording this interview, and we will be using an external transcription service to prepare a transcript of this interview. The transcription service has provided a signed confidentiality statement indicating they will not disclose any of the information from this interview. In addition, we will not collect any identifying information from you. Please do not refer yourself by name or provide any identifying information about yourself during this interview.

Before we begin, would you please confirm your consent to participate by stating,
*“Yes, I agree to participate in this interview.”*

[Wait for participant to consent. If participant does not wish to participate, please thank the participant and end the WebEx meeting].

Thank you for your consent. Are you ready to begin the interview?

Thank you, I will start the audio recording of our interview.

***Image and Title Preference***

First, we would like to ask questions about some of the images and titles you saw on the survey.

I will share my screen so that you can see them.

[Share screen with image and title].

Are you able to see the image and title?

1. Please take a look at this ***image and title***. You selected this as the image and title **you liked the most**.

- Why did you select this as the one you **liked the most**?
- What did you notice first about this? Was this **appealing to you**? Why or why not?
- Was this **unappealing to you in any way**? Why or why not?
- Was there anything else that you did not like? If yes, what did you not like? Why?
- Do you have any other comments you would like to share?

1. Please take a look at this ***image and title***. You selected this as the image and title you **liked the least**.

- Why did you select this as the one you **liked the least**?
- What did you notice first about this? Was this **not appealing to you**? Why or why not?
- Was this **appealing to you in any way**? Why or why not?
- Was there anything else that you did like? If yes, what did you like? Why?
- Do you have any other comments you would like to share?

***Science Interest and Background***

Next, we would like to ask you about your interests.

1. Do you consider yourself a science person or someone who is interested in science?

Why? Or Why not?

1. What is your prior educational background in science?
2. What are your other experiences with science?
3. Do you watch nature and wildlife shows on television? Why or why not?
4. Do you watch nature and wildlife shows on YouTube? Why or why not?
5. The images and titles you saw were for nature and wildlife videos on YouTube. Do you think you would watch these videos? Why or why not?

***Science Identity***

Finally, we would like to ask you about your identities, specifically, the identities you mentioned were most important to your sense of self or who you are.

These are the identities you listed as most important:

[State identities listed below]

[Fill in from survey responses]___________________________________________________

1. Do you think these identities are compatible with a science identity as a science person or someone who is interested in science?

Why or why not?

1. Do you have any other comments you would like to add?

Thank you so much for your time and for your assistance with this study. We have your email and we will send a gift card to you by email.
